# Object detection in histology: A multi-dataset benchmark and test-time inference

**DOI:** 10.1371/journal.pone.0354618

**Published:** 2026-07-29

**Authors:** Dragoș-Vasile Leordean, Eugen-Richard Ardelean

**Affiliations:** 1 Molecular Neuroscience Laboratory, Transylvanian Institute of Neuroscience, Cluj-Napoca, Romania; 2 UK Dementia Research Institute, Cambridge, United Kingdom; 3 Department of Computer Science, Technical University of Cluj-Napoca, Cluj-Napoca, Romania; Macau University of Science and Technology, MACAO

## Abstract

Medical image analysis has become increasingly important for automated medical diagnosis, as well as deep learning. Specifically, object detection models may help in automatically identifying pathological structures and features. This study presents a comprehensive comparative analysis for object detection tasks in histological images of the latest models including the YOLO (You Only Look Once) architectures, from YOLOv8 to the recently introduced YOLOv12. These models were evaluated alongside alternative architectures including RT-DETR, YOLO-World, and YOLOE across five diverse histology datasets: BCNB, Nuclei, TNBC, MoNuSAC, and CryoNuSeg. The experimental analysis employed standardized training protocols with consistent hyperparameters and data augmentation strategies, evaluating the performance through multiple metrics, inference time, and computational cost. The results obtained on the five datasets indicate that YOLOv11 consistently showed a strong performance across multiple datasets, however the newly introduced attention mechanisms of YOLOv12 show good performance, despite the model having slightly lower overall performance. Specialized variants like YOLOE demonstrated promising results for specific applications, while RT-DETR showed poor performance on smaller objects, which are typical in histological images. Statistical analyses indicate that YOLOv11 indeed has the best performance but that all models have a poor performance on objects of small sizes; moreover, the most common cases of failure are background false positives and missed detections. This comprehensive evaluation provides insights for the current state of object detection architectures for clinical histopathology applications and establishes benchmarks for future avenues of research in automated medical image analysis. In addition to the multi-model benchmark, we propose Test-time Graph Similarity Propagation (TGSP), a test-time self-supervised refinement that uses ResNet50 deep features to build a k-NN similarity graph over detections and performs label propagation to re-score predicted boxes. TGSP replaces TSBP’s iterative Earth-Mover matching with adaptive per-class quantile thresholds and graph-based label propagation, eliminating K-means hyperparameters and better scalability. Our analysis on histology datasets TGSP consistently matches or improves F1 relative to both a fixed 0.5 threshold and TSBP, with the biggest gains when base-model confidence calibration is poor.

## 1. Introduction

### 1.1 Histology

Human pathologists perform diagnoses by analysing, through a microscope, a treated and stained specimen [1] on a glass slide [2]. This process is called histopathology [1] while the images used in the process are called histopathological images [1]. Through specialised hardware, the entire slide may be captured with a scanner and saved as a high-resolution digital image, also called a Whole Slide Image (WSI) [2].The digitization of histopathology has revolutionized the field, enabling the development of computational approaches for automated analysis and diagnosis. To visualize different components of the tissue under a microscope, the sections are first treated and then dyed with one or more stains, with Hematoxylin and Eosin (H&E) being the most commonly used staining technique [1]. The histological images are used for the accurate diagnosis of different diseases, such as cancers, enabling the visualization of the complexity at multiple levels, enabling the analysis of intricate tissue structures, diverse cellular morphologies, and pathological changes that guide treatment decisions.

Manual annotation of histological structures is time consuming and subject to inter-variability of experts [3,4], as well as heavily reliant on expert knowledge. These challenges are a motivation for the development of automated solutions that can provide consistent, rapid and objective analysis of histological specimens. These same complexities of histological images present unique challenges for computer vision algorithms.

Databases of histological images have been made publicly available which spiked the attempt to apply deep learning techniques to automate the analysis of structures present in histological slides [3,5–7]. These deep learning approaches have shown promising results in pathology image analysis [8–11] on various tasks including cell detection, nucleus detection [12–14], tissue segmentation, and cancer diagnosis [15,16], demonstrating the potential to augment pathologist expertise, improve diagnostic accuracy and decrease the inherent time-cost.

### 1.2 Neural networks in medical applications

The research community has identified the potential of deep learning algorithms for biomedical image analysis due to their efficient processing of complex data [12,17]. The advancements in both hardware and deep learning architectures present an unprecedented potential to advance medical image analysis [12], with segmentation and object detection models showing particular promise [13,14,16] in general and for histopathological applications [8–11]. Neural networks have been increasingly used in medical applications in recent years [18,19] with newer and more performant approaches being created [30]. This is also supported by the substitution of image recognition techniques with deep learning approaches [20–22] including in pathological image analysis. Among these deep learning approaches, segmentation and object detection models have emerged as particularly useful for biomedical images analysis [12] with a three-fold increase between 2018 and 2022 as the third most popular task in medical images [19].

Several studies have demonstrated the effectiveness of object detection and segmentation models. In nucleus detection/segmentation with fluorescence microscopy, YOLO and U-Net [13] have been used with promising results. A combination of U-Net models has been developed for automatic tissue segmentation [7]. In OCT scans, U-Net and U-Net++ [23], models were used for the identification of biomarkers through the segmentation of retinal layers and fluids.

Object detection architectures can be divided into three categories: points-based detection models, two-stage detection models and single-stage detection models [24]. Perhaps the most known models throughout the large number of architectures are the “You Only Look Once” (YOLO) [24] family of models. The YOLO models have seen a great number of variants and versions through the years with new internal mechanisms for general improvements in performance or for specific tasks. Their notoriety can be attributed to their single-stage approach to detection which provides a balance between speed and accuracy, an important requirement for real-time analysis, as is the case in the biomedical field.

The YOLO architectures have been demonstrated to have applicability in a variety of medical tasks. YOLOv4 was applied for the nucleus detection in breast histopathological images [14]. YOLOv5 has shown a high performance for coronary OCT [25]. A modified YOLOv7 outperformed other traditional methods in the automated detection of blood vessels [26]. YOLOv8 and YOLOv9 have shown progress in feature detection for diabetic retinopathy from retinal fundus images with some limitations [27]. YOLOv5 and YOLOv8 were evaluated using Spectrum-Aided Visual Enhancer (SAVE) for early esophageal cancer detection by converting white-light images into hyperspectral narrowband-like images and then training on paired white-light and HSI-NBI datasets [28]; YOLOv5 was found to have the best performance through a comparison through precision, recall, F1-score, mAP, and confusion matrices [28]. YOLOv5 was found to have a strong performance for breast-lesion identification and classification for a Contrast-Enhanced Mammography study involving 1,673 patients and 7,443 images [29]. In a YOLOv5-based hyperspectral imaging study [30] for skin-cancer detection, it was shown that the hyperspectral narrowband images (HSI-NBI) can improve the recall for certain classes over the classical RGB images. YOLO-family models are also being explored in combination with spectral or contrast-enhanced imaging inputs [31,32] to improve detection and classification on the road towards clinical applicability.

Several literature reviews [1,8,33,34] have discussed the analysis of histopathological images including the use of machine learning algorithms. Machine learning has been identified as a promising avenue of research in histopathology due to its ability to uncover subtle patterns invisible to the human eye [15,35] with the potential to trace cancer origins in histopathological images [15,36]. Other studies [12] have identified the YOLO family of models as the most frequently used ranging across a wide variety of medical applications such as brain tumor detection, glaucoma detection, pulmonary embolism detection, skin lesion detection, kidney disease localization and many others [12]. The YOLO family of models was also identified as the most performant due to their robustness, high accuracy, and low computational cost [12].

YOLO has also been previously suggested and applied to histology [12]. YOLOv3 achieved a high performance in the detection of prostate cancer in histological images [37]. YOLOv4 was used in nucleus detection in breast histopathological images [14], as well as YOLOv5 [16]. A modification of YOLOv5 called Histology-based Detection using Yolo (HD-Yolo) [11] was designed for nucleus segmentation and tumor microenvironment characterization. YOLOv8 was applied for cochlea detection (using bounding boxes for localization) in macaque histology [37]. A hybrid pipeline was developed for fast cell detection using YOLOv11 and StarDist [38] for cell detection and segmessntation that was shown to be highly performant on both both proprietary data and the public MoNuSeg benchmark dataset [39].

A comparative analysis was designed to evaluate the more traditional object detection architectures based on CNNs against newer ones based on attention mechanisms. A selection of highly performant object detection models from the latest YOLO versions from YOLOv8-v12 against alternative architectures such as RT-DETR, YOLO-World, YOLOE were evaluated. These models were evaluated on five diverse histological datasets from the perspective of five performance metrics (mAP@50, mAP@50–95, F1 score, precision and recall) and computational efficiency (inference time, FLOPs). A standardized parametrization was used in the analysis to guarantee a fair comparison including hyperparameter optimization and data augmentation.

Our contributions include: (1) benchmarking of the latest YOLO versions from YOLOv8-v12 against alternative architectures such as RT-DETR, YOLO-World, YOLOE across five diverse datasets for object detection of cancerous regions in histopathological images, (2) an analysis of performance trade-offs between accuracy, speed, and computational efficiency in the clinical context of the data, (3) statistical analysis to determine detection capability, distribution of failures and statistical ranking, (4) a graph-based method for test-time inference refinement and its performance evaluation and (5) insights for the deployment of automated histology analysis systems in clinical settings.

## 2. Materials and methods

### 2.1 Datasets

We selected five histology datasets to comprehensively evaluate the performance of object detection models with a focus on breast tissue images with H&E staining, yet datasets including other organs have been included as well. All annotation types have been converted to YOLO bounding boxes to ensure compatibility with the object detection paradigm and consistent evaluation across all models. The datasets represent a range of clinical scenarios and technical challenges commonly encountered in computational analysis of histopathological data. A small description relating the characteristics of each dataset is shown in Table 1.

**Table 1:** Short description of datasets.

Dataset	Organs	Staining	Annotation type	Image count	Classes
BCNB [40]	Breast	H&E	JSON polygon vertices	1058	1
Nuclei [10]	Breast	H&E	PNG binary mask	143	1
TNBC [42]	Breast	H&E	PNG binary mask	50 (from 11 patients)	1
MoNuSAC [43]	Breast Lung Prostrate Kidney	H&E	XML region vertices	209	4 names: [Epithelial, Lymphocyte, Neutrophil, Macrophage]
CryoNuSeg [41]	Adrenal gland Larynx Lymph nodes Mediastinum Pancreas Pleura Skin Testes Thymus Thyroid gland	H&E	PNG binary mask	30 (3 per organ)	1

Scalebars are not included in the histological images presented given that: 1) the images have previously been published, as indicated, and are used for feature detection and 2) we were unable to obtain information regarding the magnification of the images in two of the datasets (BCNB [40] and CryoNuSeg [41]).

#### 2.1.1 BCNB.

**Fig 1:**
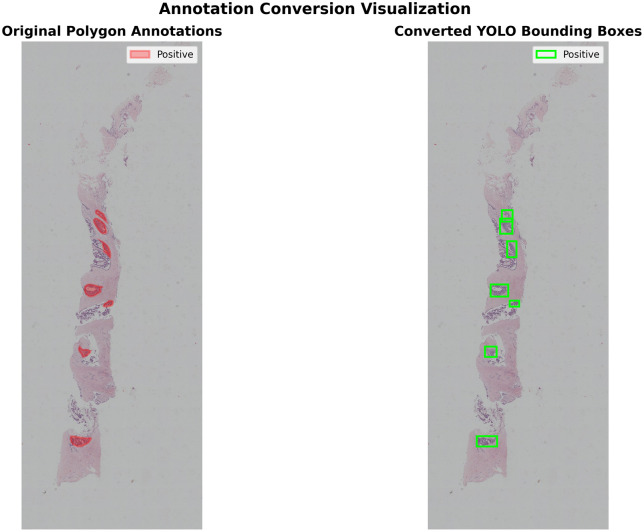
Example from the BCNB [40] dataset with the original annotations (left) and the converted yolo bounding boxes (right).

BCNB [40] dataset comprises digitized H&E-stained WSIs of primary tumor core-needle biopsy (CNB) specimens for predicting axillary lymph node (ALN) metastasis in early breast cancer (EBC). This extensive collection includes data from 1,058 EBC patients with clinically negative ALN, enrolled between May 2010 and August 2020. To ensure the dataset’s quality and relevance for deep learning applications, strict inclusion criteria were applied, such as pathologically confirmed primary invasive breast cancer, comprehensive baseline clinicopathological data, and adequate biopsy material. The dataset was systematically divided, with 840 patients allocated to the training cohort and 218 patients to an independent test cohort, alongside a validation cohort comprising 25% of the training data. The JSON-based polygons have been automatically converted to bounding boxes for the object detection models. An example of conversion can be found in Fig 1.

Due to the large size of the WSI histological images, a compression must be made to increase the efficiency and reduce both the storage size and inference time of neural network models [44]. Downsampling of 16x has been shown to improve the precision of object detection models while slightly lowering recall on renal WSI histological images [44], with optimal downsampling being at 8x. Compression is another available option for further downsizing the high resolution images [44], with an optimal rate of 40%. Furthermore, lower resolution images have been shown to beneficially impact performance of segmentation models [45]. All images have been downsampled 20x due to their large size and computational cost.

#### 2.1.2 Nuclei.

**Fig 2:**
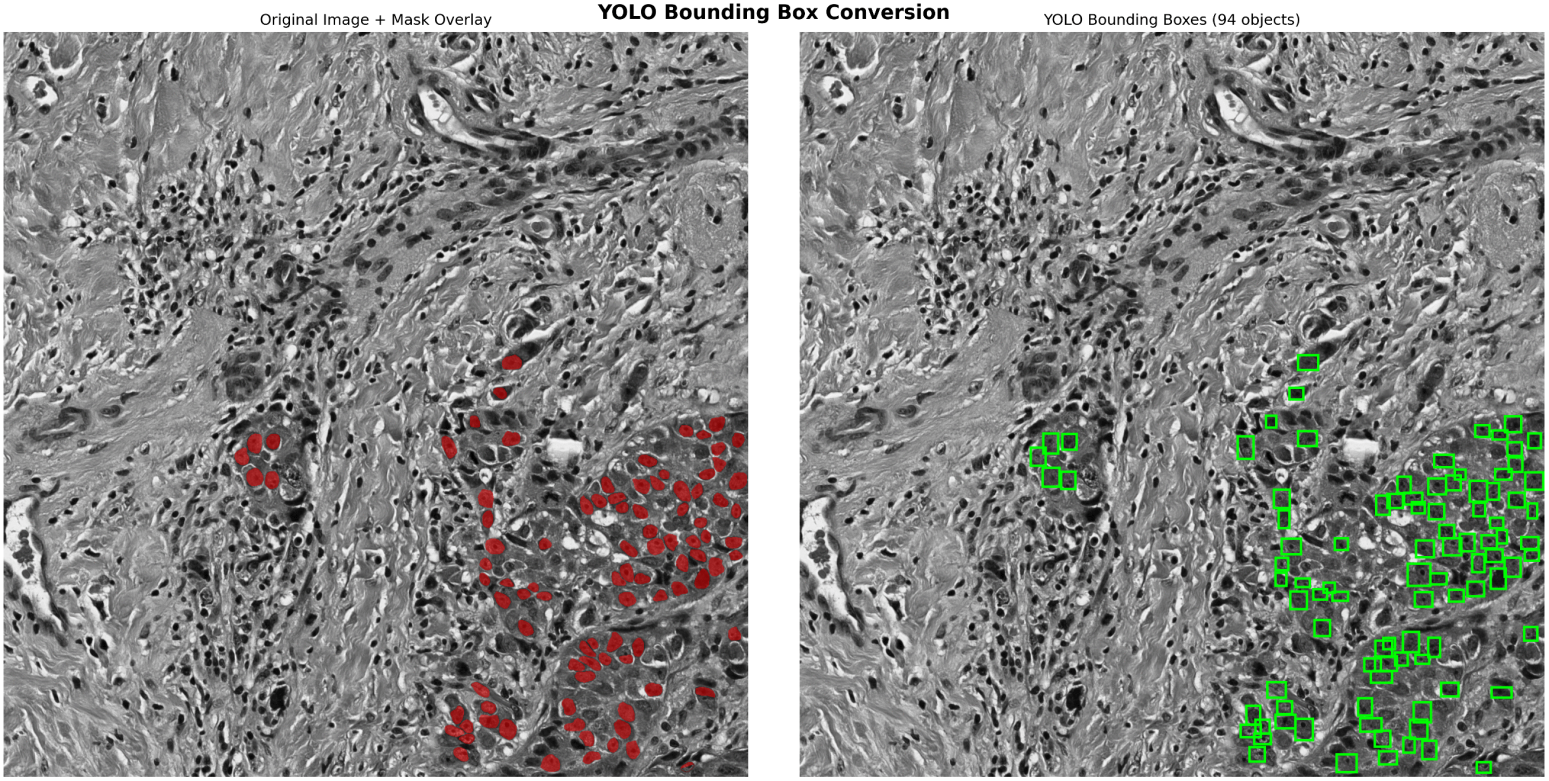
Example from the Nuclei [10] dataset with the original annotations (left) and the converted yolo bounding boxes (right).

Nuclei [10] dataset comprises 143 2,000 x 2,000 images ER + BCa images scanned at 40x with 12,000 nuclei manually segmented across 137 patients. The segmentation masks have been automatically converted to bounding boxes for the object detection models. An example of conversion can be found in Fig 2.

#### 2.1.3 TNBC.

**Fig 3:**
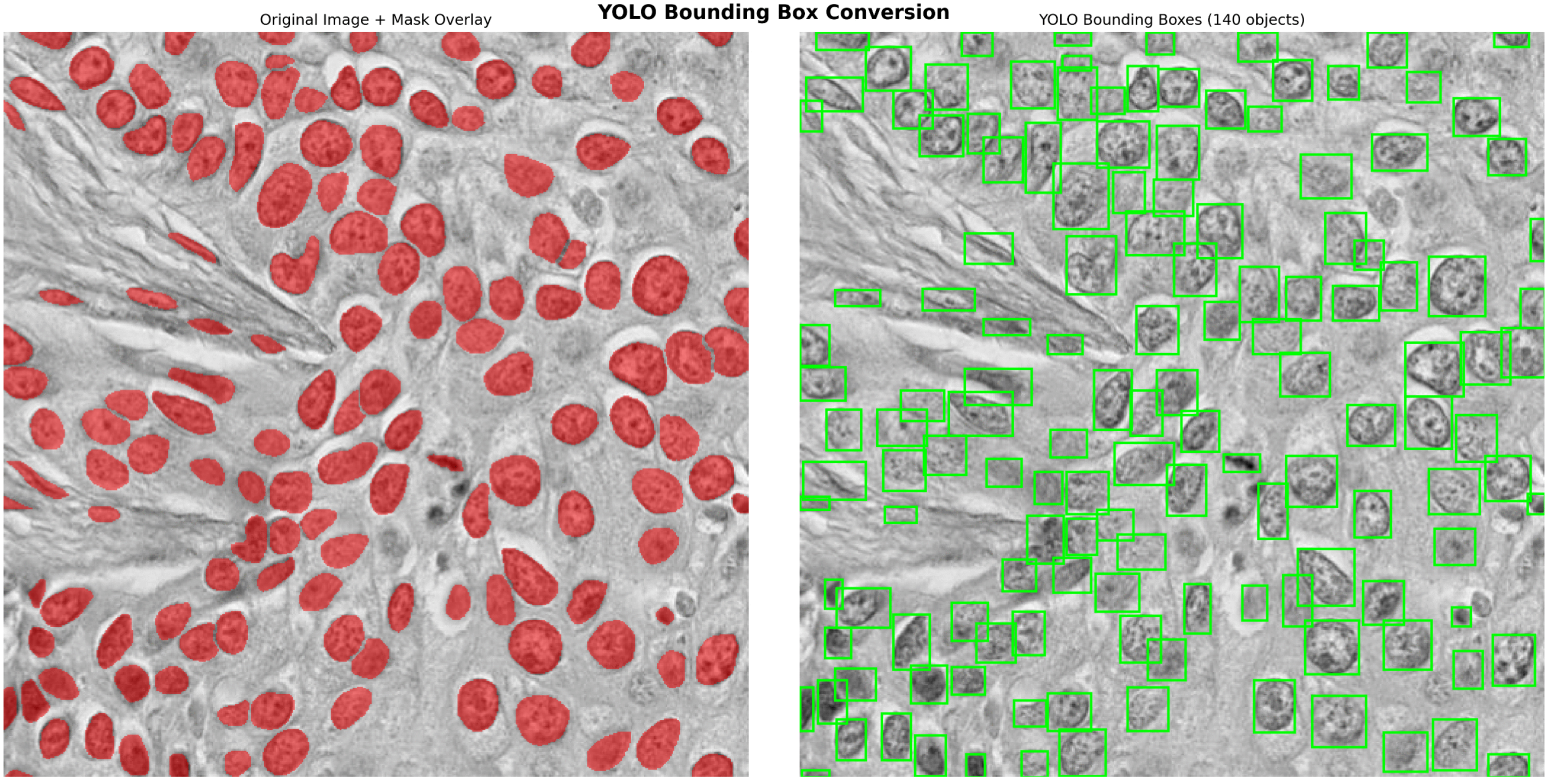
Example from the TNBC [42] dataset with the original annotations (left) and the converted yolo bounding boxes (right).

TNBC [42] dataset was generated at the Curie Institute, comprising 50 images from 11 patients with a total of 4,022 annotated cells from H&E stained histological slides at 40x magnification. These slides were sourced from Triple Negative Breast Cancer (TNBC) patients. The segmentation masks have been automatically converted to bounding boxes for the object detection models. An example of conversion can be found in Fig 3.

#### 2.1.4 MoNuSAC.

**Fig 4:**
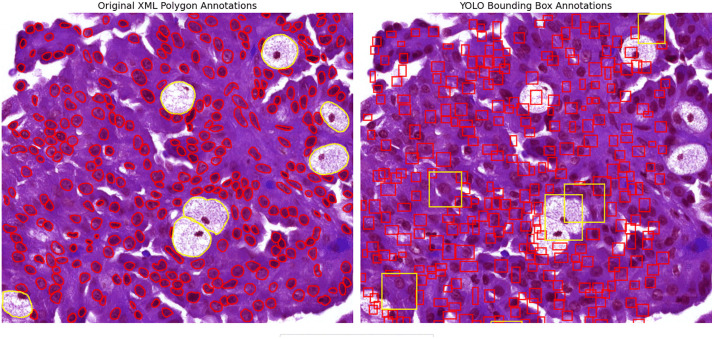
Example from the MoNuSAC [43] dataset with the original annotations (left) and the converted yolo bounding boxes (right).

The MoNuSAC [43] dataset is a comprehensive, multi-organ dataset specifically designed for nuclei segmentation in histopathological images to encourage the computer vision research community to develop and test algorithms for detecting, segmenting, and classifying nuclei, tasks crucial for characterizing the tumor microenvironment (TME) in cancer prognostication and research. This large and diverse dataset comprises over 46,000 hand-annotated nuclei, sourced from 71 patients across 31 hospitals. It includes nuclei from four different organs and four distinct nucleus types: epithelial cells, lymphocytes, macrophages, and neutrophils. The annotations were reviewed by an expert pathologist, with iterative revisions ensuring less than 1% error in annotations. The XML-based polygons have been automatically converted to bounding boxes for the object detection models. An example of conversion can be found in Fig 4.

#### 2.1.5 CryoNuSeg.

**Fig 5:**
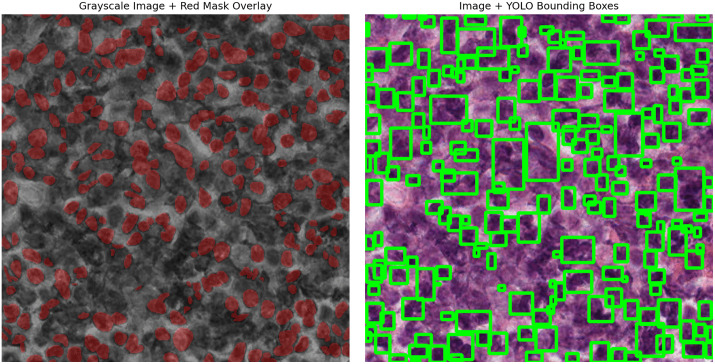
Example from the CryoNuSeg [41] dataset with the original annotations (left) and the converted yolo bounding boxes (right).

CryoNuSeg [41] is manually annotated nuclei instance segmentation dataset derived exclusively from frozen section (FS) H&E-stained images. CryoNuSeg comprises images from 10 human organs that were not exploited in other publicly available datasets, specifically the adrenal gland, larynx, lymph node, mediastinum, pancreas, pleura, skin, testis, thymus, and thyroid gland. It includes 30 whole slide images (WSIs), from which 512x512 pixel image patches acquired at 40x magnification were extracted, with one patch per WSI. The binary segmentation masks have been automatically converted to bounding boxes for the object detection models. An example of conversion can be found in Fig 5.

### 2.2 Object detection models

The “You Only Look Once” (YOLO) [24] algorithm revolutionized real-time object detection through the unification of region proposal and classification. YOLO divides an image through a grid attempting to predict a class and a bounding box for each cell of the grid. This approach significantly reduced computation time and enabled end-to-end learning. YOLOv1 managed to achieve real-time detection although it incorporated a CNN backbone; however, small objects were hard to identify and localize accurately due to the fact that each grid cell was only able to predict two bounding boxes.

Since the introduction of YOLO in 2015, the YOLO family of algorithms [46] has expanded both in depth and width with several incremental improvements and various modified versions for specific applications. Each new version introduced advancements with the goal of improving computational efficiency and detection accuracy. YOLOv2 [46–48] brought several enhancements to YOLOv1 including higher input resolution, batch normalization, and the adoption of anchor boxes from Faster R-CNN to improve training and performance. YOLOv3 [46,47,49] achieved higher accuracy in small-object detection through multi-scale predictions using Feature Pyramid Networks (FPN) and a deeper architecture, specifically Darknet-53. YOLOv4 [46,47,50] managed to set new standards for detection accuracy and inference speed through several new introductions, such as: CSPDarknet-53, PANet, Mish activation, and data augmentation methods such as Mosaic and CutMix. YOLOv5 by Ultralytics [46,47,51] was designed with a PyTorch-based framework with an emphasis on usability and performance and made several changes such as: enhanced backbone, neck, and head designs, and an auto-anchor learning mechanism that optimized anchor sizes during training. YOLOv6 [46,47,52] added the EfficientRep backbone and RepOptimizer to improve efficiency. YOLOv7 [46,47,53] improved efficiency by introducing a trainable bag-of-freebies and the Extended Efficient Layer Aggregation Network (E-ELAN).

YOLOv8 [46,47,51], also from Ultralytics [51], integrated the C2f module into the architecture and transitioned to anchor-free detection for a more simplified and specialized detection. YOLOv9 [46,47,54] incorporated the Generalized Efficient Layer Aggregation Network (GELAN) and Programmable Gradient Information (PGI) to mitigate information degradation in deep networks. YOLOv10 [46,47,55] removed non-maximum suppression (NMS) and introduced a dual assignment strategy and lightweight classification heads achieving real-time efficiency. YOLOv11 [46,47,51] integrated the C3k2 block, SPPF, and C2PSA into the architecture to improve feature extraction and spatial attention while balancing precision and efficiency.

YOLOv12 [46,47,51,56] shifted toward an attention-centric architecture achieving state-of-the-art accuracy and efficiency while maintaining real-time performance [47,56]. The innovations that allowed YOLOv12 to achieve this (confirmed through ablation studies [56]) are: the Area Attention module, which reduces computational complexity while maintaining a large receptive field, and Residual Efficient Layer Aggregation Networks (R-ELAN) that enhance feature aggregation and optimize training for larger models. Other architectural updates include FlashAttention for memory optimization, MLP ratio adjustments, attention-convolution integration for efficiency, removal of positional encoding, and the Position Perceiver using large separable convolutions for positional awareness. Extensive analyses confirmed YOLOv12’s performance [56] showing increased accuracy, speed, and reduced computational cost compared to YOLOv6, YOLOv8, YOLOv10, YOLOv11, and RT-DETR variants. Despite its recent introduction in February 2025 [56], YOLOv12 has been successfully applied to early detection of sexually transmitted diseases [57], UAV tracking of [58], fruit detection [59,60], and marine litter detection [61,62] outperforming earlier YOLO versions.

YOLO-World [63] was derived from YOLOv8 and it added open-vocabulary detection using vision-language modeling demonstrating increased performance in real-world applications and in zero-shot scenarios for a wide range of objects. YOLO-World allows for the interaction between visual and linguistic information through the Re-parameterizable Vision-Language Path Aggregation Network (RepVL-PAN) and the alignment of this information through region-text contrastive loss, enhancing zero-shot detection for diverse applications.

YOLOE [64] was derived from YOLOv8 and YOLOv11 and it added prompt-based mechanisms for both object detection and instance segmentation which allows for the integration of contextual information; thus, enhancing preprocessing with context-relevant information [65]. It supports text prompts via the Re-parameterizable Region-Text Alignment (RepRTA) strategy, visual prompts via the Semantic Activated Visual Prompt Encoder (SAVPE), and no prompts via the Lazy Region-Prompt Contrast (LRPC) strategy.

Real-Time DEtection TRansformer (RT-DETR) [60,66,67] is an alternative approach to object detection based on an end-to-end transformer removing the need for non-maximum suppression. RT-DETR replaces the standard Transformer encoder with a hybrid encoder to process intra-scale interactions and cross-scale feature fusion separately, increasing speed and detection accuracy via a query selection mechanism. RT-DETRv2 [67] achieves increased multi-scale detection through several modifications: dynamic data augmentation, scale-adaptive hyperparameters, and Selective Multi-Scale Sampling.

The YOLO family of models are released in multiple model scales offering trade-offs between accuracy, speed, computational cost, and parameter count. They are represented by suffixes: nano (n), small (s), medium (m), large (l), extra large (x). Larger models provide higher accuracy at increased computational cost, allowing users to select versions based on specific application needs, from maximum speed (n) to highest precision (x).

### 2.3 Performance evaluation

Precision represents the percentage of positive predictions that are correct and is calculated as the ratio of true positives (TP) to the sum of true positives and false positives (FP). Precision measures the ability to avoid false alarms and is computed as:


$$\begin{array}{c} Precision=\frac{TP}{TP+FP} \end{array}$$

Where *TP* represents the correct detections compared to the ground truth and *FP* represents the incorrect detections where no object actually exists.

Recall represents the percentage of actual positives correctly identified and is calculated as the ratio of true positives to the sum of true positives and false negatives (FN). Recall measures the ability to avoid missed detections and is computed as:


$$\begin{array}{c} Recall=\frac{TP}{TP+FN} \end{array}$$

Where *TP* represents the correct detections compared to the ground truth and *FN* represents the cases where an object is missed compared to the ground truth.

The F1 score is considered a balanced measure of performance as it computed as the harmonic mean of precision and recall. In contrast to precision and recall, the F1 score incorporates both for false positives and false negatives. This makes it particularly useful for the evaluations of performance in applications where correctly identifying positive cases is as important as minimizing false alarms. It is computed as:


$$\begin{array}{c} F1=\frac{2*Precision*Recall}{\left(Precision+Recall\right)} \end{array}$$

Intersection over Union (IoU) represents the overlap in area between predicted and true bounding boxes. IoU assesses how accurately the model is able to capture object boundaries and is computed as:


$$\begin{array}{c} IoU=\frac{Intersectionarea}{Unionarea} \end{array}$$

Mean Average Precision at an IoU threshold of 0.5 (mAP@50) represents the area under the precision-recall curve, also known as the Average Precision (AP), computed for each class where detections achieve an overlap with with true bounding boxes at least 0.5 IoU. It is computed as:


$$\begin{array}{c} mAP@50=\frac{1}{\left|C\right|}\sum_{c\in C}{AP}_{c}(IoU\geq 0.5) \end{array}$$

Where *c* represents a single class of from the set of classes *C*.

Mean Average Precision across IoU thresholds from 0.5 to 0.95 (mAP@50–95) represents AP scores averaged across multiple IoU thresholds ranging from 0.5 to 0.95 in increments of 0.05. This metric reflects localization performance under varying strictness levels, penalizing detections that only loosely match true objects. It is computed as:


$$\begin{array}{c} mAP@50-95=\frac{1}{\left|C\right|}\sum_{c\in C}\left(\frac{1}{\left|T\right|}\sum_{t\in T}{AP}_{c}(IoU\geq t)\right) \end{array}$$

Where *c* represents a single class of from the set of classes *C*, and *t* represents a single threshold from the set of thresholds *T* ={0.5, 0.55,..., 0.95}.

Although commonly practiced, evaluating performance using a single performance metric can be risky endeavor [68,69]. Especially if the metric fails to capture all relevant aspects, potentially leading to misleading and biased results. Therefore, we employed multiple complementary metrics to provide a more thorough evaluation across different aspects of performance.

### 2.4 Test-time inference

Fixed global confidence thresholds are commonly used to decide which bounding boxes to keep during test-time inference [70]. However, this approach introduces a brittle tradeoff of precision and recall as high thresholds will reduce false positives but also remove true positives, while low thresholds will introduce more false positives. Most commonly, an evaluation is made on the validation set across different thresholds to determine the deployment fixed confidence threshold [70,71]. Object detection tasks involve diverse arrays of objects of different sizes and shapes and may include multiple imbalanced classes for which a fixed threshold may not be optimal. Moreover, the training dataset is unlikely to include the whole spectrum of characteristics possible within a certain domain; thus, as more data is accumulated slight differences in distribution can appear that will require a change in the threshold [72,73]. Statistically, predictions that have received a higher confidence score are more likely to be correct detections than those that received a lower confidence score [70].

Methods have been developed to recalibrate the raw confidence scores obtained, such as Platt scaling [74], histogram binning [75], Bayesian binning [76] and Beta calibration [77]. The major disadvantage of these methods is the requirement of held-out labelled data to estimate parameters. Moreover, these methods remain sensitive to the same limitations as those of fixed thresholds [70].

The Test-time Self-guided Bounding-box Propagation (TSBP) [70] is a novel method designed to determine which objects are to be kept by detection models in a test-time scenario without the use of labelled data. TSBP determines which of the lower confidence predictions are valid by using visual similarity to the “confirmed” higher confidence predictions. It segregates them into high-confidence “confirmed” sets (termed true positive predictions that are above a confidence threshold of 0.5), uncertain candidate set based on specific thresholds and low-confidence set (termed false positive predictions that are below a confidence threshold of 0.3). As the predicted bounding boxes are of different sizes, it rescales them before extracting features through a ResNet-50 model to obtain features to determine the visual similarities between predictions. TSBP applies K-means clustering to the high-confidence predictions to establish the initial distance constraints derived from the average shortest Euclidean distance between these confirmed detections.

TSBP performs a multi-round Earth Mover’s Distance (EMD) matching process to determine which lower-confidence candidates are visually similar to the higher confidence “confirmed” predictions by minimizing the feature-space distance. This propagation process from higher confidence predictions to candidates happens in two stages: first it employs strict distance constraints to ensure more precise label assignments, second it relaxes these constraints to allow for a broader range of candidates.

TSBP determines visual similarity on the test set at inference time requiring no additional labeled samples or parameter estimation and managed to obtain higher F1 scores on two datasets compared to baseline thresholding and Logic Calibration [70,74,78], Beta Calibration [70,77,78] and Histogram Binning [70,75,78].

As mentioned above, higher confidence scores are more likely to be correct; however, this does not mean that a high confidence value was obtained. A first weakness of TSBP is that it considers true positive detections above a fixed threshold of 0.5. This global threshold approach of TSBP may not be optimal across different datasets, classes, or other detection scenarios where the distribution of confidence scores can vary. Moreover, TSBP relies on K-means clustering to obtain representative samples from both high-confidence and low-confidence detections, which introduces additional hyperparameters that can require manual tuning as low values may not capture detection diversity while high values can introduce noise.

We propose Test-time Graph Similarity Propagation (TGSP), a graph-based approach inspired by TSBP that leverages label propagation on similarity graphs rather than iterative Earth Mover’s Distance (EMD) matching.

Unlike TSBP’s fixed confidence thresholds, TGSP employs adaptive per-class thresholds based on the score distribution of each class. Instead of fixed thresholds on confidence values, adaptive thresholds are created based on quantiles for high-confidence and low-confidence predictions on each class. This adaptive approach automatically adjusts to the confidence distribution of each class in the test set, eliminating the need for manual threshold tuning and better accommodating datasets with varying detection difficulty. Moreover, the per-class approach allows for a fair partition when class imbalanced detections (such as a class with a low number of detections per image and another with a high number) are found as each class will receive high-confidence and low-confidence predictions.

TGSP replaces TSBP’s iterative EMD matching with a graph-based label propagation. For all N detections, *k*-nearest neighbor (*k*-NN) graph is constructed where each detection is represented as a node, edges connect each detection to its *k* most similar neighbors based on feature distance and edge weights are computed using a Gaussian kernel (using the Euclidean distance between feature vectors). Features are computed identically to TSBP through the use of a ResNet50 model.

Using the adaptive thresholds, a label matrix Y ∈ ℝ^N×2^ is initialized obtaining a soft-label assignment representation of the initial confidence values for detections:


$$\begin{array}{c} mAP@50-95=\frac{1}{\left|C\right|}\sum_{c\in C}\left(\frac{1}{\left|T\right|}\sum_{t\in T}{AP}_{c}(IoU\geq t)\right) \end{array}$$

Iteratively labels are propagated using:


$$\begin{array}{c} Y^{t+\text{1}}=\alpha \cdot S{\cdot Y}^{t}+(\text{1}-\alpha )\cdot Y^{\text{0}} \end{array}$$

Where $Y^{t}$ represents the label distribution at iteration *t*, α∈[0,1] controls the balance between neighborhood influence and initial labels. Through this process, confidence information is propagated from labeled (high/low confidence) detections to unlabeled ones through the similarity graph. The propagation continues until convergence or when the change in *Y* falls below a threshold. After propagation is finished, each detection has two scores representing its TP and FP likelihoods. A detection is accepted as a true positive if the TP is larger than the FP

Both TSBP and TGSP are self-supervised in the sense that only the test set predictions are used and do not require additional training or labelled data. Both use deep features obtained from ResNet-50 to measure visual similarity between detections and both use a similarity propagation approach to improve low-confidence detection by leveraging high-confidence detections. However, TGSP may be faster by replacing the multi-round EMD optimization with a time complexity of *O(N³)* in worst case with a *O(N log N· d)* graph construction, while also minimizing a graph-based function which offers smooth transitions and is a non-greedy approach that considers global graph structure.

## 3. Results

### 3.1 Performance on individual datasets

All models were trained using identical experimental conditions to ensure fair comparison. The training procedure used the same random subset of 70% of the original dataset for training, while validation and testing subsets contained 20% and 10%, respectively (except for the TNBC datasets in which splits were made by patients, and the CryoNuSeg dataset with splits by organs). All results presented are on the previously-unseen non-augmented test sets.

Models were trained for 50 epochs across the entire training set with batches of 16 images rescaled to 640 × 640 pixels. The AdamW optimizer was used with a starting learning rate of 1e-4, which was reduced to 1e-6 during the training process. A small penalty to large weights was added through a weight decay of 5e-4. A training learning rate warmup of 10 epochs was used to stabilize training. Data augmentation was applied to reduce the possibility of overfitting. Training images in HSV color space were randomly adjusted with hue, saturation, and value shifts of 0.015, 0.7, and 0.4, respectively. Additional augmentations included random rotations of up to ±10°, translations of up to 10% along either axis, and random zoom variations of up to 20%. Mixup augmentation was applied during training, blending two images into a single composite to enhance generalization by introducing additional noise and variability. Mosaic augmentation was also employed to combine multiple images, simulating visually complex scenarios. For model stability, mosaic augmentation was disabled during the final 10 training epochs.

The comparative evaluation of object detection models across five histology datasets showed considerable variations in performance depending on the dataset characteristics and models. Highlighting importance of considering multiple evaluation metrics and dataset- and domain-specific challenges when selecting object detection models for specific applications. The comparative evaluation was performed on the previously unseen non-augmented test partitions of the datasets.

#### 3.1.1 BCNB.

The BCNB dataset [40], containing 1058 breast cancer nuclei images, presented a challenge for all evaluated models as shown in Table 2, presumably due to the small object sizes as this dataset contains the most images. YOLOv12 achieved the highest mAP@50 score of 0.285, closely followed by YOLOv10 with 0.281. However, the performance gap between different YOLO versions was relatively narrow, suggesting that the dataset characteristics may be too complex even for the advanced features of newer architectures.

**Table 2:** Results of object detection models on the BCNB [40] dataset (across 5 runs presented as mean±standard deviation, bold values indicate the best performance for each criterion).

Model/Metric	mAP@50	mAP@50–95	Mean F1	Mean Precision	Mean Recall	Inference Time [ms]	Computational Cost [GFLOPs]
RT-DETR	0.181 ± 0.003	0.083 ± 0.002	**0.262 ± 0.002**	0.267 ± 0.004	**0.257 ± 0.002**	1010.3	103.4
YOLOv8	0.249 ± 0.020	0.131 ± 0.006	0.177 ± 0.027	0.424 ± 0.056	0.114 ± 0.026	210.9	28.4
YOLOv9	0.259 ± 0.010	0.135 ± 0.009	0.198 ± 0.037	0.431 ± 0.043	0.131 ± 0.035	205.8	26.7
YOLOv10	0.265 ± 0.018	0.143 ± 0.009	0.158 ± 0.047	**0.462 ± 0.064**	0.099 ± 0.038	**126.7**	24.5
YOLOv11	0.261 ± 0.006	0.142 ± 0.005	0.242 ± 0.006	0.405 ± 0.007	0.172 ± 0.005	148.2	21.3
YOLOv12	**0.279 ± 0.007**	**0.155 ± 0.000**	0.215 ± 0.013	0.460 ± 0.000	0.141 ± 0.011	150.2	**21.2**
YOLOv8-Worldv2	0.256 ± 0.005	0.142 ± 0.001	0.197 ± 0.007	0.428 ± 0.003	0.128 ± 0.006	169.6	32.6
YOLOE	0.259 ± 0.021	0.135 ± 0.016	0.242 ± 0.025	0.395 ± 0.071	0.182 ± 0.043	135.6	35.3

The results show that RT-DETR performed poorly across all metrics, with particularly low mAP scores and extremely high inference times (1010.3 ms), making it unsuitable for real-time clinical applications. YOLOv10 demonstrated the best speed-accuracy trade-off, achieving high precision (0.265) with the fastest inference time (126.7 ms) among YOLO models despite its slightly higher computational cost of 24.5 GFLOPs, though at the cost of lower recall (0.099). YOLOv12, with its attention mechanisms, showed the best performance, suggesting that this dataset may benefit from the increased architectural complexity. The computational cost remained comparable between YOLOv11 and YOLOv12 (21.3 vs 21.2 GFLOPs), but YOLOv12 showed slightly higher inference time.

**Fig 6:**
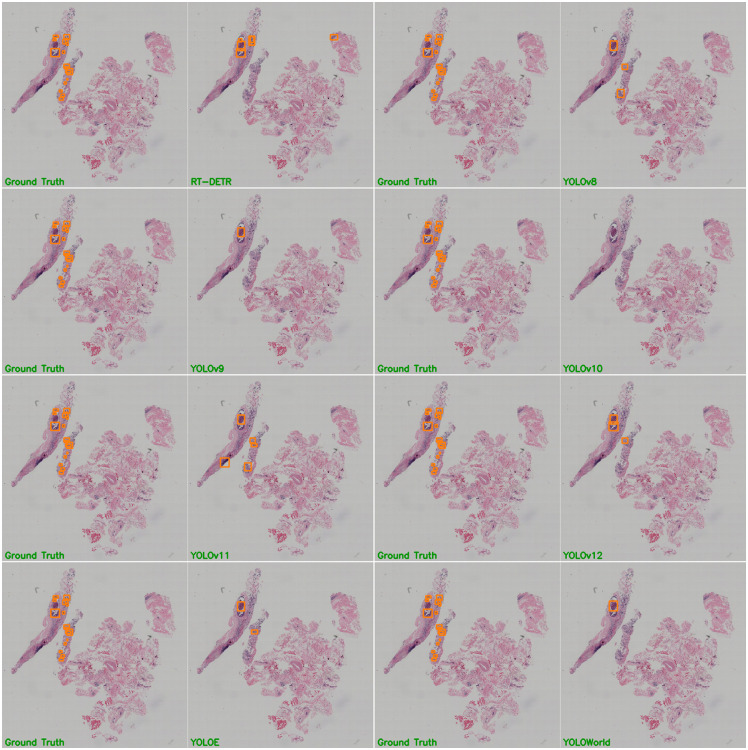
Example from the BCNB [40] dataset with the true annotations (left) and the predicted bounding boxes (right). The results suggest that the models are unable to comparably recapitulate the bounding boxes observed in the ground truth.

The visual inspection of results shown in Fig 6 suggests that none of the models were capable of learning the correlations between the bounding boxes and the small-scale features of the images. With a subset of models introducing false positives that are not localized near the pathological features, or even not being able to predict any bounding boxes for certain images.

The impact of downsampling was analyzed with various amounts on the YOLO11 model for the BCNB dataset. The performance for each level of downsampling is shown in Table 3 indicating that more downsampling (x40) would reduce the performance of the model, whilst less downsampling (x8, x16) may bring a slight increase in performance. Nevertheless, with lower downsampling the images have a higher resolution requiring a bigger model which has a higher training and inference time. Therefore, the reduced performance obtained by all models on this dataset cannot be attributed to the downsampling.

**Table 3:** Results of YOLO11 object detection model on the BCNB [40] dataset (across 5 runs presented as mean±standard deviation, bold values indicate the best performance for each criterion).

Down sampling	mAP@50	mAP@50–95	Mean F1	Mean Precision	Mean Recall	Inference Time [ms]
x8	**0.268 ± 0.003**	**0.149 ± 0.004**	0.262 ± 0.005	0.404 ± 0.004	0.194 ± 0.005	207.6
x16	0.263 ± 0.002	0.148 ± 0.006	**0.268 ± 0.006**	0.392 ± 0.005	**0.204 ± 0.008**	172.5
x20	0.261 ± 0.006	0.142 ± 0.005	0.242 ± 0.006	**0.405 ± 0.007**	0.172 ± 0.005	148.2
x40	0.216 ± 0.016	0.109 ± 0.011	0.128 ± 0.012	0.385 ± 0.034	0.077 ± 0.010	121.4

#### 3.1.2 Nuclei.

The Nuclei dataset [10], containing only 143 images, presented unique challenges related to limited training data and small object sizes as shown in Table 4. The results showed more pronounced differences between models, with some architectures failing completely; nevertheless, the detection accuracy is higher than for the previous dataset.

**Table 4:** Results of object detection models on the Nuclei [10] dataset (across 5 runs presented as mean±standard deviation, bold values indicate the best performance for each criterion).

Model/Metric	mAP@50	mAP@50–95	Mean F1	Mean Precision	Mean Recall	Inference Time [ms]	Computational Cost [GFLOPs]
RT-DETR	0.00 ± 0.00	0.00 ± 0.00	0.00 ± 0.00	0.00 ± 0.00	0.00 ± 0.00	1123.8	103.4
YOLOv8	0.401 ± 0.026	0.234 ± 0.014	0.374 ± 0.022	**0.557 ± 0.040**	0.282 ± 0.017	301.8	28.4
YOLOv9	0.372 ± 0.040	0.219 ± 0.031	0.418 ± 0.023	0.442 ± 0.094	0.418 ± 0.080	211.9	26.7
YOLOv10	0.388 ± 0.029	0.234 ± 0.013	0.390 ± 0.022	0.512 ± 0.052	0.318 ± 0.027	**141.3**	24.5
YOLOv11	0.417 ± 0.015	0.242 ± 0.001	0.442 ± 0.026	0.487 ± 0.037	0.411 ± 0.061	155.6	21.3
YOLOv12	0.381 ± 0.111	0.235 ± 0.098	0.425 ± 0.078	0.425 ± 0.169	**0.471 ± 0.049**	187.7	**21.2**
YOLOv8-Worldv2	0.405 ± 0.009	0.246 ± 0.012	0.422 ± 0.019	0.531 ± 0.026	0.352 ± 0.038	212.2	32.6
YOLOE	**0.438 ± 0.030**	**0.260 ± 0.022**	**0.466 ± 0.009**	0.512 ± 0.063	0.433 ± 0.030	150.9	35.3

RT-DETR completely failed on this dataset, achieving zero performance across all metrics. This catastrophic failure suggests that this transformer-based architecture may require more training data or may not be well-suited for the small object sizes typical in histological detection tasks.

YOLOE achieved the highest mAP@50 score of 0.438 and F1 of 0.466, closely followed by YOLOWorld and YOLOv11. The prompt-guided capabilities of YOLOE appeared to provide advantages on this limited dataset, possibly by better leveraging contextual information during training.

Interestingly, YOLOv12 underperformed compared to YOLOv11, achieving only 0.381 mAP@50. However, YOLOv12 showed the highest recall (0.471), suggesting it performed better at detecting all nuclei, but with lower precision. This trade-off indicates that YOLOv12’s attention mechanisms may be capable of detecting more potential objects, but with reduced discrimination capability on this small dataset.

**Fig 7:**
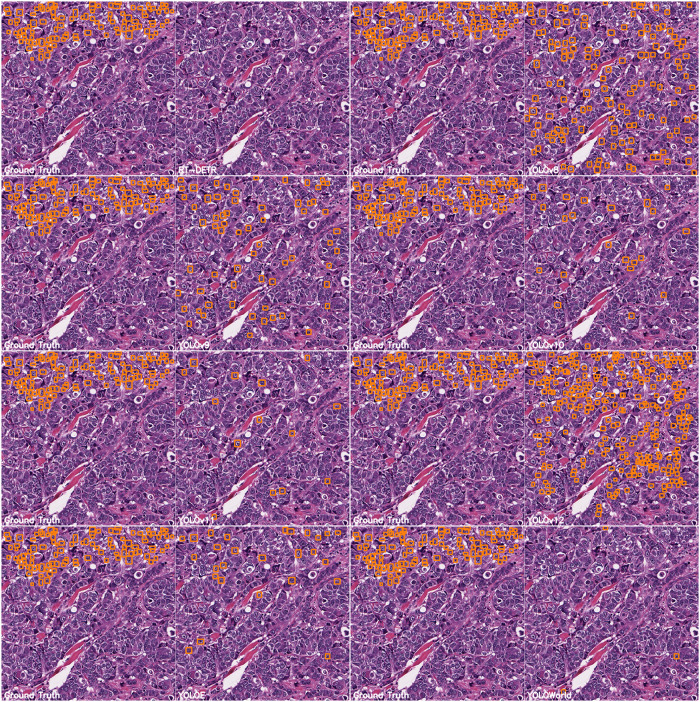
Example from the Nuclei [10] dataset with the true annotations (left) and the predicted bounding boxes (right). The results suggest that the models are unable to comparably recapitulate the bounding boxes observed in the ground truth (either due to a significant number of false positives, such as YOLO12, or due to a lack of predictions, such as YOLOW).

Certain insights can also be gained from the visual inspection of results shown in Fig 7. The lower performance of YOLOv12 may be attributed to its tendency to over-predict resulting in many false positives. In contrast, YOLOv11 and YOLOE produce fewer predictions that align more closely the true bounding boxes.

#### 3.1.3 TNBC.

The TNBC dataset [42] was split across patients into the train, validation and test sets. The 11 patients were split as follows: patients 01–09 into the train set, patient 10 into the validation set and patient 11 into the test set. The chosen splitting of the dataset allowed for an experimental setup where the cross-patient generalization of these models could be evaluated.

The results showed exceptionally high performance across most YOLO models, indicating that triple-negative breast cancer nuclei are relatively well-characterized and distinguishable. Moreover, despite the small number of examples in the dataset, the high performance of models indicates that object size might be a more important factor than the dataset size. The results obtained by each model are presented in Table 5.

**Table 5:** Results of object detection models on the TNBC [42] dataset (across 5 runs presented as mean±standard deviation, bold values indicate the best performance for each criterion).

Model/Metric	mAP@50	mAP@50–95	Mean F1	Mean Precision	Mean Recall	Inference Time [ms]	Computational Cost [GFLOPs]
RT-DETR	0.244 ± 0.163	0.130 ± 0.087	0.282 ± 0.189	0.181 ± 0.121	0.641 ± 0.429	1193.6	103.4
YOLOv8	0.920 ± 0.005	0.653 ± 0.003	0.898 ± 0.004	0.929 ± 0.018	0.869 ± 0.010	315.0	28.4
YOLOv9	**0.936 ± 0.007**	0.615 ± 0.021	0.898 ± 0.015	0.901 ± 0.010	**0.895 ± 0.020**	229.8	26.7
YOLOv10	0.890 ± 0.002	0.639 ± 0.003	0.862 ± 0.002	0.899 ± 0.018	0.829 ± 0.011	177.7	24.5
YOLOv11	0.926 ± 0.004	**0.677 ± 0.006**	0.904 ± 0.005	**0.937 ± 0.001**	0.874 ± 0.009	**167.6**	21.3
YOLOv12	0.930 ± 0.002	0.674 ± 0.003	**0.909 ± 0.002**	0.932 ± 0.011	0.887 ± 0.006	189.5	**21.2**
YOLOv8-Worldv2	0.672 ± 0.263	0.440 ± 0.241	0.677 ± 0.227	0.741 ± 0.208	0.626 ± 0.236	241.1	32.6
YOLOE	0.922 ± 0.003	0.602 ± 0.001	0.904 ± 0.005	0.936 ± 0.017	0.874 ± 0.005	170.7	35.3

YOLOv9 achieved the highest mAP@50 scores of 0.936, demonstrating excellent detection capability. However, YOLOv11 achieved the highest mAP@50–95 score of 0.677, indicating superior localization accuracy across stricter IoU thresholds. This suggests that YOLOv11’s architecture provides better boundary delineation, which is crucial for accurate quantitative analysis of nuclear morphology. YOLOv12 also achieved the highest F1 score (0.909) and recall (0.887), indicating the best overall balance between precision and recall and a relatively low inference speed of only 189.5 ms, making it a possible choice for real time applications.

RT-DETR showed improved performance on this dataset compared to other datasets, but still significantly underperformed compared to YOLO architectures. The extremely high recall (0.641) but low precision (0.181) suggests RT-DETR was over-detecting, producing many false positives.

**Fig 8:**
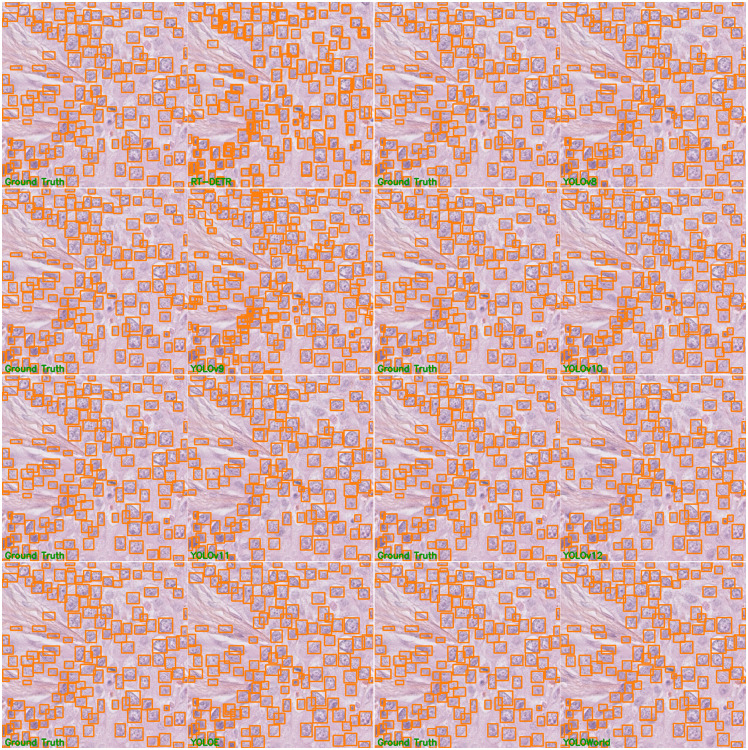
Example from the TNBC [42] dataset with the true annotations (left) and the predicted bounding boxes (right). The results suggest that all models are able to identify with high accuracy the true bounding boxes with a low number of missed boxes.

The visual inspection shown in Fig 8 corroborates the insights extracted from the performance metrics. RT-DETR does indeed predict false positives in areas that do not contain pathological features, whilst YOLOv12 has the most accurate bounding boxes. The other YOLO models whilst predicting somewhat accurate bounding boxes tend to predict multiple overlapping boxes for pathological features and miss smaller areas or areas that blend in with the background.

To confirm that these results are not due to the patient split and that true generalization can occur, a new analysis was created. In Table 6, the results of the YOLO11 model are presented for 11 different splits (including new rounds of training and testing from scratch) where the images of each patient sequentially become the previously unseen test set (TNBC-01 indicates that the model was trained on images excluding patient 01 and tested on patient 01). The results indicate that regardless of split, the YOLO11 is capable of a high amount of generalization showing an average mAP@50 performance of ~0.9 with minimum variation of ±0.03 depending on the split.

**Table 6:** Results of the YOLO11 object detection models on the TNBC [42] dataset for various splits into train, validation and test subsetss (across 5 runs presented as mean±standard deviation, bold values indicate the best performance for each criterion).

Model/Metric	mAP@50	mAP@50–95	Mean F1	Mean Precision	Mean Recall
TNBC-01	**0.929 ± 0.001**	0.626 ± 0.017	0.890 ± 0.007	0.898 ± 0.018	0.882 ± 0.005
TNBC-02	**0.929 ± 0.005**	0.625 ± 0.011	0.887 ± 0.008	0.915 ± 0.033	0.861 ± 0.014
TNBC-03	0.928 ± 0.014	0.642 ± 0.010	0.895 ± 0.007	0.915 ± 0.016	0.877 ± 0.028
TNBC-04	0.867 ± 0.006	0.596 ± 0.003	0.834 ± 0.008	0.869 ± 0.011	0.802 ± 0.010
TNBC-05	0.894 ± 0.005	0.556 ± 0.022	0.851 ± 0.019	0.834 ± 0.034	0.868 ± 0.006
TNBC-06	0.856 ± 0.033	0.558 ± 0.008	0.824 ± 0.016	0.837 ± 0.017	0.812 ± 0.025
TNBC-07	0.926 ± 0.003	0.529 ± 0.017	0.897 ± 0.009	0.901 ± 0.032	**0.893 ± 0.018**
TNBC-08	0.891 ± 0.003	0.582 ± 0.017	0.831 ± 0.008	0.823 ± 0.017	0.840 ± 0.002
TNBC-09	0.915 ± 0.015	0.641 ± 0.012	0.866 ± 0.010	0.883 ± 0.015	0.849 ± 0.018
TNBC-10	0.879 ± 0.026	0.591 ± 0.015	0.846 ± 0.021	0.861 ± 0.017	0.834 ± 0.055
TNBC-11	0.926 ± 0.004	**0.677 ± 0.006**	**0.904 ± 0.005**	**0.937 ± 0.001**	0.874 ± 0.009

#### 3.1.4 MoNuSAC.

The MoNuSAC dataset [43], with its multi-organ, multi-class detection task, provided the most comprehensive evaluation of model capabilities across diverse tissue types and cellular morphologies. The results across models are shown in Table 7.

**Table 7:** Results of object detection models on the MoNuSAC [43] dataset (across 5 runs presented as mean±standard deviation, bold values indicate the best performance for each criterion).

Model/Metric	mAP@50	mAP@50–95	Mean F1	Mean Precision	Mean Recall	Inference Time [ms]	Computational Cost [GFLOPs]
RT-DETR	0.306 ± 0.000	0.069 ± 0.000	0.230 ± 0.000	0.449 ± 0.000	0.203 ± 0.000	1010.4	103.4
YOLOv8	0.771 ± 0.027	**0.506 ± 0.021**	0.716 ± 0.025	0.785 ± 0.007	0.676 ± 0.042	**190.2**	28.4
YOLOv9	0.764 ± 0.023	0.482 ± 0.022	0.717 ± 0.020	**0.816 ± 0.005**	0.656 ± 0.036	208.4	26.7
YOLOv10	0.714 ± 0.019	0.447 ± 0.007	0.662 ± 0.018	0.743 ± 0.030	0.619 ± 0.037	238.8	24.5
YOLOv11	0.752 ± 0.040	0.491 ± 0.032	0.701 ± 0.040	0.808 ± 0.003	0.637 ± 0.062	211.5	21.3
YOLOv12	0.757 ± 0.042	0.505 ± 0.032	0.705 ± 0.045	0.770 ± 0.056	**0.688 ± 0.014**	206.3	**21.2**
YOLOv8-Worldv2	0.770 ± 0.012	0.489 ± 0.007	0.705 ± 0.016	0.788 ± 0.026	0.671 ± 0.033	199.6	32.6
YOLOE	**0.779 ± 0.007**	0.450 ± 0.004	**0.721 ± 0.012**	0.803 ± 0.004	0.685 ± 0.001	253.0	35.3

YOLOE achieved the highest mAP@50 score of 0.779, demonstrating the advantage of attention mechanisms for multi-class detection tasks. YOLOE achieved the third-highest mAP@50 (0.757) and the highest mAP@50–95 (0.505), indicating excellent performance across both detection and localization followed closely by YOLOv11 and YOLO-World. YOLOE demonstrated strong performance with the highest F1 score (0.721) and competitive recall (0.671), suggesting that prompt-guided detection may be particularly beneficial for distinguishing between different cell types. RT-DETR again showed poor performance, particularly in mAP@50–95 (0.069), indicating severe localization problems.

**Fig 9:**
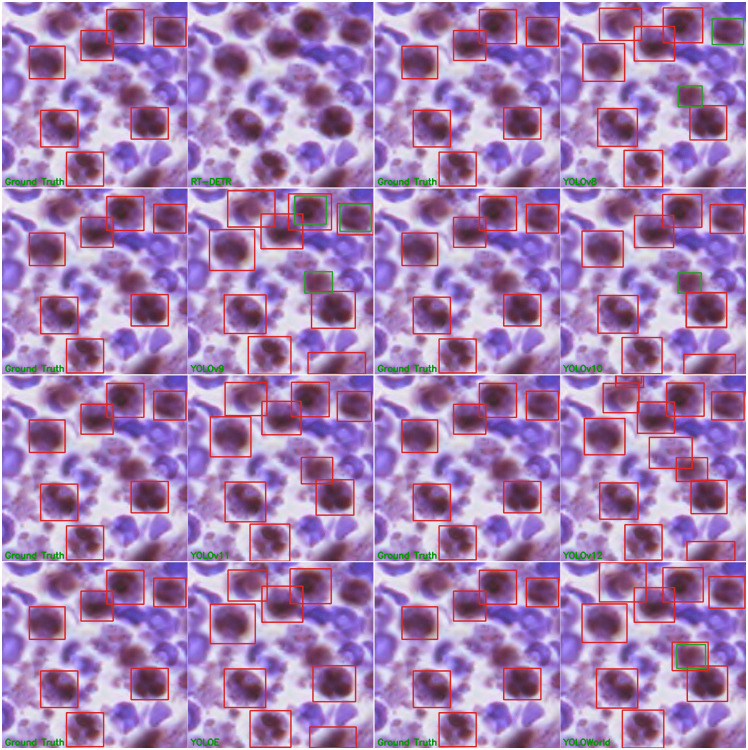
Example from the MoNuSAC [43] dataset with the true annotations (left) and the predicted bounding boxes (right). The results suggest that all models (except RT-DETR) are able to identify with high accuracy the true bounding boxes; nevertheless, there is a set of annotations that are missed (such as the top-right annotation in YOLOE) or false positives of a different class (such as the green boxes in YOLO8/ YOLO9/ YOLO10/ YOLOW).

The visual inspection shown in Fig 9 demonstrates that all models have wrong predictions, specifically they produce false positives in areas with similar features to those of true pathological areas. Moreover, several models predicted classes that are not present in the images even overlapping with correct predictions.

#### 3.1.5 CryoNuSeg.

As the CryoNuSeg dataset [41] offers 10 different organs, the dataset was split by organs for training, validation and testing. All organs but the adrenal gland and the larynx have been used for training, whilst the adrenal gland was used for validation and the larynx for testing. The chosen splitting of the dataset allowed for an experimental setup where the cross-organ generalization of these models could be evaluated. The results of this experiment are shown in Table 8.

**Table 8:** Results of object detection models on the CryoNuSeg [41] dataset (across 5 runs presented as mean±standard deviation, bold values indicate the best performance for each criterion).

Model/Metric	mAP@50	mAP@50–95	Mean F1	Mean Precision	Mean Recall	Inference Time [ms]	Computational Cost [GFLOPs]
RT-DETR	0.031 ± 0.000	0.014 ± 0.000	0.072 ± 0.000	0.056 ± 0.000	0.104 ± 0.000	778.9	103.4
YOLOv8	0.773 ± 0.000	0.463 ± 0.007	0.741 ± 0.004	0.742 ± 0.018	0.740 ± 0.009	**169.4**	28.4
YOLOv9	0.747 ± 0.005	0.455 ± 0.003	0.714 ± 0.005	0.709 ± 0.024	0.721 ± 0.020	170.2	26.7
YOLOv10	0.682 ± 0.008	0.445 ± 0.011	0.677 ± 0.010	0.689 ± 0.006	0.666 ± 0.026	186.9	24.5
YOLOv11	**0.784 ± 0.003**	**0.503 ± 0.004**	**0.756 ± 0.007**	**0.745 ± 0.026**	**0.768 ± 0.013**	216.2	21.3
YOLOv12	0.745 ± 0.032	0.446 ± 0.039	0.721 ± 0.020	0.725 ± 0.024	0.716 ± 0.019	179.0	**21.2**
YOLOv8-Worldv2	0.377 ± 0.435	0.210 ± 0.242	0.362 ± 0.418	0.373 ± 0.430	0.352 ± 0.407	186.9	32.6
YOLOE	0.750 ± 0.001	0.432 ± 0.008	0.727 ± 0.013	0.715 ± 0.008	0.740 ± 0.034	241.2	35.3

YOLOv11 achieved the highest mAP@50 score of 0.784 and the highest mAP@50–95 of 0.503, demonstrating superior performance for this cross-organ detection task. YOLOv11 also achieved the best balance between precision (0.756) and recall (0.745), resulting in the highest F1 score (0.762). YOLOv11 was closely followed by YOLOv8, YOLOv9, YOLOv12 and YOLOE on all metrics. YOLOv8 achieved the second-highest mAP@50 (0.773) with the fastest inference time among all models (169.4 ms), demonstrating that earlier YOLO versions can still provide competitive performance on certain tasks. RT-DETR showed catastrophic failure on this dataset with extremely low performance across all metrics (mAP@50 of 0.031), suggesting fundamental incompatibility with the small object sizes.

**Fig 10:**
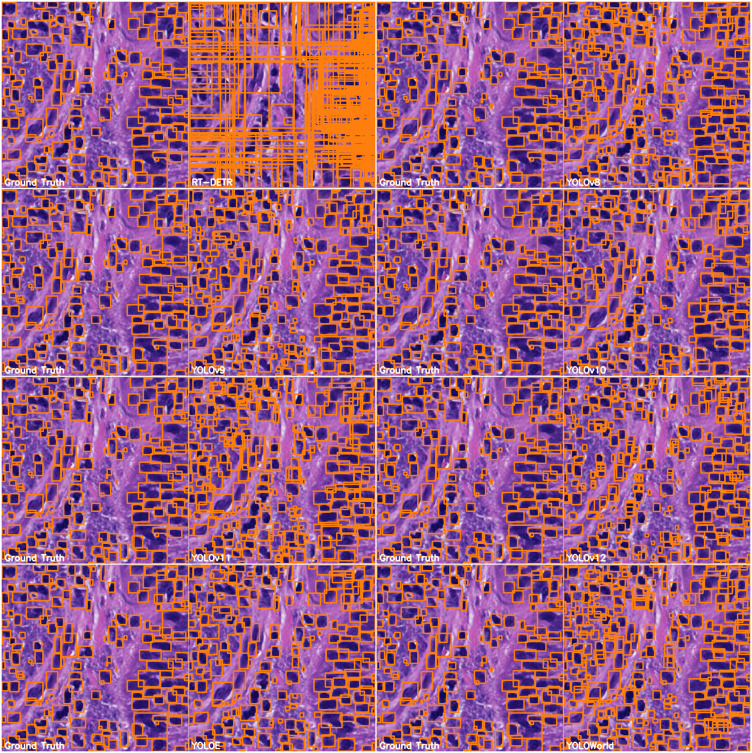
Example from the CryoNuSeg [41] dataset with the true annotations (left) and the predicted bounding boxes (right). The results suggest that all models (except RT-DETR) are able to identify with high accuracy the true bounding boxes; nevertheless, there are several cases of overlapping boxes which can be attributed to the low value of the IoU-based NMS parameter.

Through the data presented in Fig 10, RT-DETR is evidently unable to correctly localize the pathological areas creating much larger boxes than required. Nevertheless, all models tend to over-predict with even smaller areas being identified as pathological although they do not contain such features.

**Fig 11:**
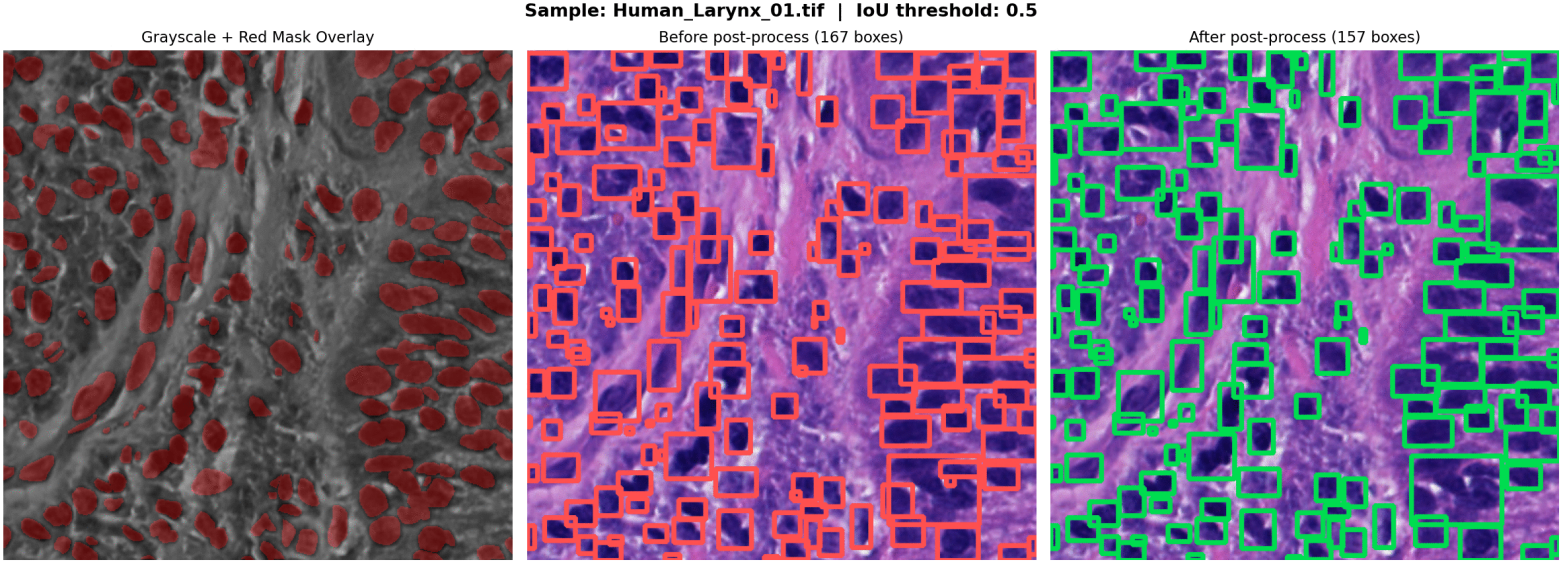
Post-processing removal of bounding boxes to avoid inclusion and heavy overlap.

Due to the high number of bounding boxes that are overlapping, especially included within each other, we have made another analysis to see whether the removal of these bounding boxes would help increase the model’s performance. The removal post-process was achieved through a hard thresholding on the intersection over union and intersection over smaller (IoS) as shown in Fig 11. In average, 5.46 bounding boxes were removed per image. Table 9 shows the impact of removal of these bounding boxes with a slight decrease in overall performance of the model, indicating that these overlapping and included bounding boxes may help in discrimination.

**Table 9:** Results of the YOLO11 object detection models on the CryoNuSeg [41] dataset with all bounding boxes and with only the bounding boxes that do not present a high overlap (across 5 runs presented as mean±standard deviation, bold values indicate the best performance for each criterion).

Model/Metric	mAP@50	mAP@50–95	Mean F1	Mean Precision	Mean Recall
Bounding boxes with no heavy overlap or inclusion	0.741 ± 0.051	0.446 ± 0.057	0.680 ± 0.105	0.648 ± 0.169	0.742 ± 0.027
All bounding boxes	**0.784 ± 0.003**	**0.503 ± 0.004**	**0.756 ± 0.007**	**0.745 ± 0.026**	**0.768 ± 0.013**

### 3.2 Statistics across datasets

**Fig 12:**
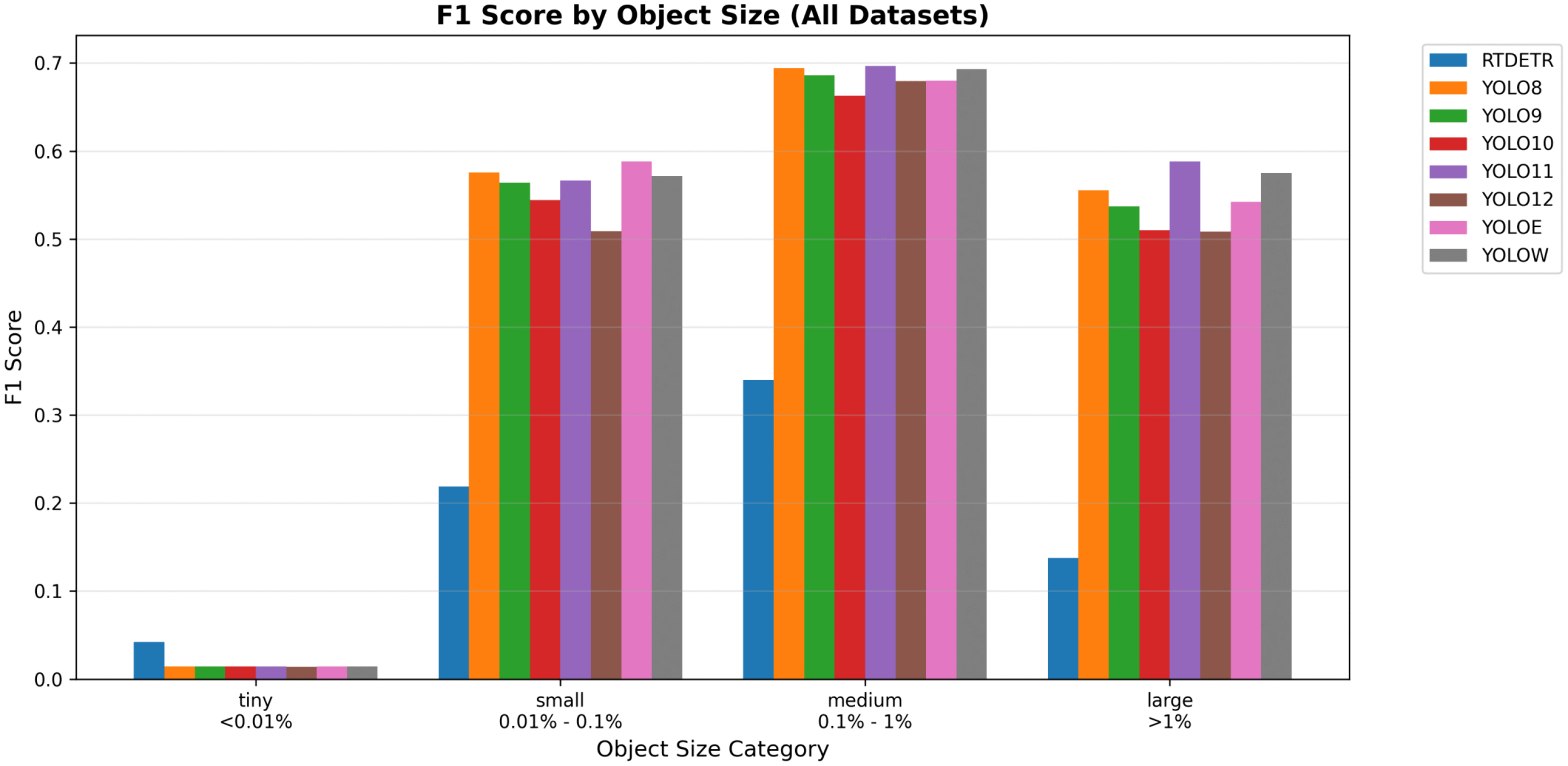
Detection accuracy (F1 score) of each model across all datasets split by object size.

To determine the capability of models to accurately detect objects based on their size, an aggregated analysis was made across all datasets and split by the size of the true bounding boxes. The true bounding boxes have been split into 4 categories based on the covered image area by percentage: tiny (<0.01% of image area), small (0.01–0.1%), medium (0.1–1%), and large (>1%). The F1-score was computed by matching predicted bounding boxes to ground truth annotations using an Intersection over Union (IoU) threshold of 0.5 where true positives were defined as predictions correctly matched to ground truth objects of the same class, false positives as unmatched predictions, and false negatives as unmatched ground truth objects. This analysis reveals how detection accuracy varies with object size, allowing for the identification of whether models struggle with smaller objects that are more challenging to detect or larger objects that may exhibit greater morphological variability. Fig 12 clearly shows that overall, the YOLO models are more capable of detecting the true bounding boxes than the RT-DETR model; however, all models are incapable of identifying objects of small sizes.

**Fig 13:**
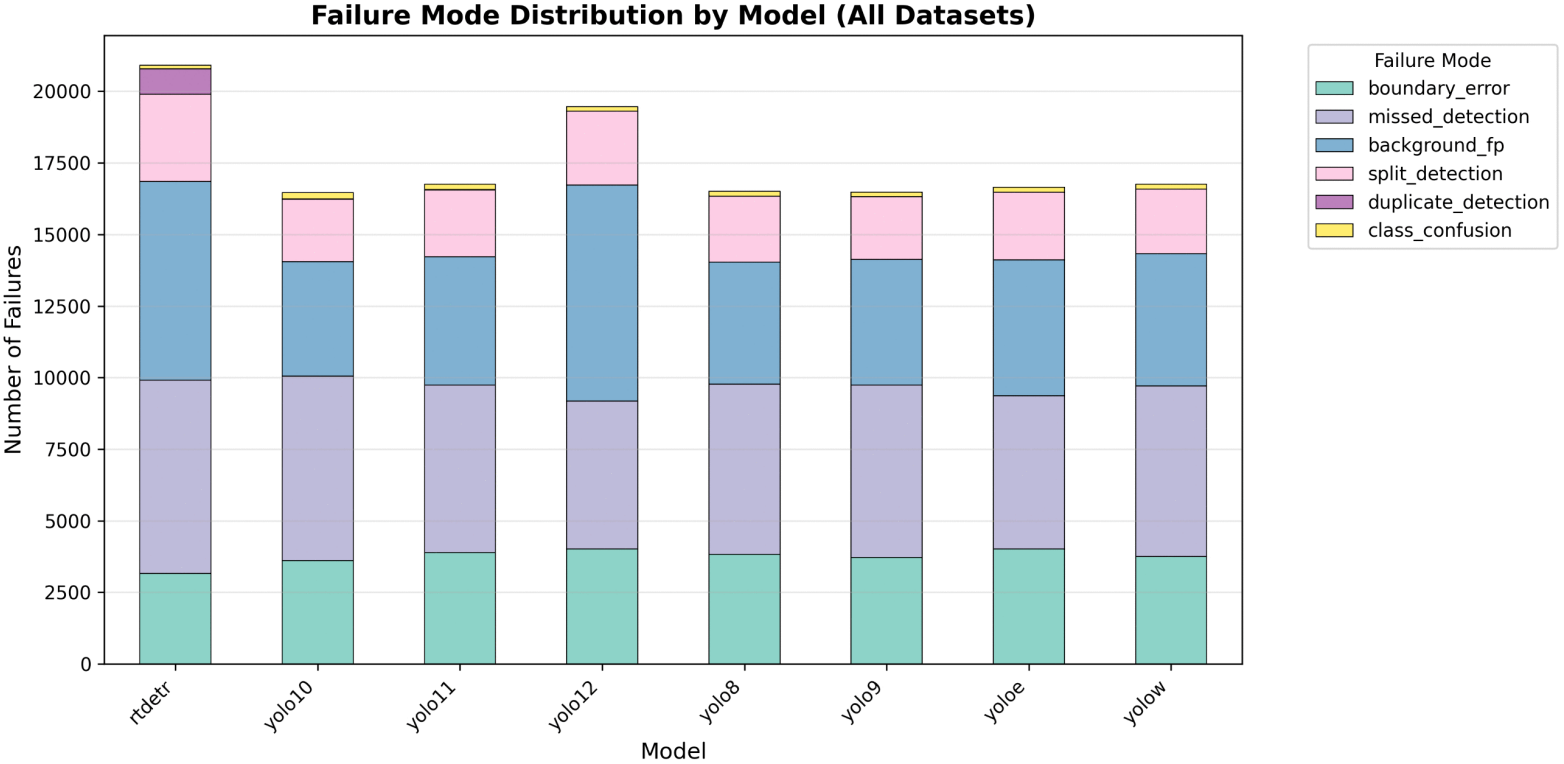
Distribution of failure cases for each model across all datasets.

To characterize the types of detection errors produced by each model, a failure mode distribution was computed. Failure modes were classified into six categories: missed detections (ground truth objects with no matching prediction above IoU threshold), boundary errors (predictions matched to ground truth but with IoU below threshold), class confusion (predictions overlapping ground truth but with incorrect class), duplicate detections (multiple predictions matching the same ground truth object), split detections (false positives overlapping with ground truth, suggesting over-segmentation), and background false positives (predictions with minimal overlap to any ground truth object). For each model across all test images of all datasets, the number of each failure type was counted, and their relative proportions were computed. This analysis allows for the identification of the dominant error type of each model, distinguishing between models that tend to miss objects entirely versus those that detect objects but with poor localization or false positive predictions. Fig 13 shows the distribution of failure cases for each model showing that most common errors are boundary errors, split detections, background false positives. The RT-DETR model had a considerably larger number of background false positives and was the only model with duplicate detections. YOLO12 also had a large number of background false positives but it compensated with the lowest number of missed detections. Specifically, the high number of background false positives observed for YOLOv12 are confined to the nuclei dataset and should be interpreted as a dataset-specific failure case rather than a general limitation of the proposed attention-centric design.

**Fig 14:**
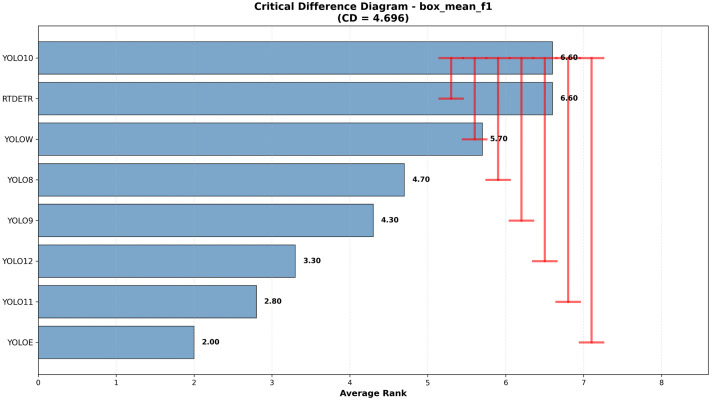
Average ranks of detection models across the five datasets and critical differences.

To assess the statistical significance of performance differences across the five datasets, Friedman test followed by Nemenyi post-hoc analysis was employed. The Friedman test was used to evaluate whether model performance (mean value across 5 runs) differed significantly across five datasets (p < 0.05 threshold). Average ranks for each model across datasets were computed and applied the Nemenyi post-hoc test to determine the critical difference (CD) threshold at α = 0.05. Models whose average rank difference exceeds this CD value are considered significantly different, while those within the CD threshold show no statistically significant performance distinction. In the critical difference diagram shown in Fig 14, models are ordered by average rank (lower is better), and vertical lines connect groups of models that are not significantly different from each other, providing a clear visual summary of the statistical equivalence relationships among competing architectures. Clearly, the best by rank is YOLOv11.

### 3.3 Test-time inference

In our analysis of test-time inference methods, three methods have been evaluated: baseline (a fixed global confidence threshold of 0.5), TSBP (using the parametrization of the authors [70]) and TGSP (the proposed method). All methods operated at test time without access to additional labeled data or model retraining and with fixed parameters across all datasets and models.

Table 10 presents the F1 scores obtained by each of these methods on each dataset when compared to the test subset ground truth clearly showing that the proposed method outperforms TSBP in almost all cases (except 2), while also giving a better performance on most combinations of datasets and models than the fixed threshold.

**Table 10:** Comparative analysis of test-time inference methods based on F1 score performance (bold values indicate cases in which the proposed method TGSP outperforms TSBP).

	CryoNuSeg	CryoNuSeg	CryoNuSeg	TNBC	TNBC	TNBC	nuclei	nuclei	nuclei
model	Baseline	TSBP	TGSP	Baseline	TSBP	TGSP	Baseline	TSBP	TGSP
RT-DETR	0.053	0.063	0.052	0.000	0.000	**0.355**	0.000	0.000	0.000
YOLOv8	0.749	0.704	**0.706**	0.855	0.833	**0.878**	0.097	0.111	**0.410**
YOLOv9	0.715	0.682	**0.690**	0.841	0.780	**0.859**	0.114	0.282	**0.429**
YOLOv10	0.532	0.595	**0.635**	0.830	0.801	**0.866**	0.063	0.162	**0.360**
YOLOv11	0.705	0.671	**0.683**	0.791	0.790	**0.878**	0.081	0.206	**0.397**
YOLOv12	0.600	0.552	**0.673**	0.797	0.774	**0.897**	0.332	0.332	0.304
YOLOv8-Worldv2	0.692	0.590	**0.694**	0.811	0.791	**0.870**	0.062	0.203	**0.401**
YOLOE	0.641	0.666	**0.691**	0.855	0.840	**0.887**	0.182	0.330	**0.456**

The variable performance increase depending on the model used suggests that the effectiveness of test-time refinement depends critically on the initial confidence calibration of the base model. Well-calibrated models may not benefit substantially, while poorly calibrated models may be beyond recovery through post-processing alone. The considerable increase in performance for TGSP on the TNBC dataset for the RT-DETR model suggests that adaptive thresholding may be more effective at recovering usable detections from severely miscalibrated models.

Both TSBP and TGSP use deep features obtained from a ResNet-50 pretrained model which renders their processing time dependent upon the hardware of the system. As such, both add a considerable amount of time to the inference. These approaches can only be used as an offline post-processing step as they do not offer real-time performance.

## 4. Conclusions

The evaluation across five histology datasets with diverse characteristics and limitations revealed several insights regarding the performance characteristics of different object detection architectures for histopathological applications. The limited training data, the small object sizes and multi-organ multi-class nature are challenges for models even without considering cross-patient and cross-organ generalization.

The high performances of all models on the experimental setup of the TNBC dataset (based on the train-validation-test split) indicate that these object detection models may be capable of cross-patient generalization as the overall performance of the YOLO models have reached a mAP@50 of ~0.9. At the same time, the results obtained through the experimental setup of the CryoNuSeg dataset (based on the train-validation-test split) indicate that these object detection models may even be capable of cross-organ generalization due to the high performance of YOLO models of ~0.75 mAP@50. All YOLO models had similar performances on the chosen datasets, yet a few conclusions about their overall ranking can be deduced from the data presented.

YOLOv11 emerged as the most consistent performer across datasets, achieving top or near-top performance on four out of five datasets. Its architecture incorporating C3k2 blocks, SPPF, and C2PSA modules proved effective for histological data. The model showed stability across object sizes and tissue types. YOLOv11 demonstrated through its performance the highest robustness on the CryoNuSeg dataset in a cross-organ generalization setup. YOLOv11 also showed a high generalization on our 11-fold patient analysis on the TNBC dataset with an average mAP@50 performance of ~0.9. This claim is further enforced by the rank analysis showing the best performance for this model.

YOLOv12, despite its attention mechanism and state-of-the-art performance on natural datasets, showed variable performance on histological images. While it achieved the highest performance on the TNBC dataset (mAP@50–95: 0.676) and competitive results on BCNB and CryoNuSeg, it underperformed on the Nuclei and MoNuSAC datasets (when compared to other YOLO architectures). This suggests that the attention-centric design may require larger training datasets or specific domain adaptations to fully leverage its capabilities for the small cellular structures typical in histology.

YOLOv10 demonstrated competitive inference times while maintaining reasonable accuracy. YOLOv8 and YOLOv9 showed solid performance across datasets, with YOLOv9 achieving top performance on TNBC (mAP@50: 0.930). These models represent well-optimized architectures that benefit from extensive development and refinement.

YOLOE demonstrated strong performance, particularly on the small Nuclei dataset (mAP@50: 0.464) and multi-class MoNuSAC dataset (mAP@50: 0.773). The prompt-guided detection capabilities appear to provide advantages for scenarios with limited training data or complex classification requirements. YOLOv8-Worldv2 showed particular strength on the multi-class MoNuSAC dataset (mAP@50: 0.780), demonstrating the value of open-vocabulary detection capabilities for applications requiring classification of diverse cell types without extensive retraining.

RT-DETR showed the lowest performance across all datasets, with catastrophic failures on the Nuclei and CryoNuSeg datasets (mAP@50: 0 and 0.031, respectively). The transformer-based architecture appears fundamentally unsuitable for the small object sizes and limited training data of histological images. The extremely high inference times (778.9–1193.6 ms) further limit its clinical applicability. YOLO architectures (126.7–315.0 ms) demonstrated 3–10 times faster inference compared to RT-DETR, while achieving significantly higher detection accuracy.

Overall, based on the similar performances of models across datasets (except RT-DETR), an empirical observation is that object sizes are a better predictor of performance than dataset size. This can be inferred from the significantly lower performance of all models on the BCNB (1058 images) and Nuclei (143 images) datasets, while at the same time the models had a high performance on the other datasets (30, 50 and 209 images) which had considerably lower dataset sizes. This observation is further enforced by the statistical analysis showing that the lowest F1 scores were obtained for the tiny subset of objects.

We introduce TGSP, a graph-based approach inspired by TSBP for refining object detection for test-time inference. TGSP replaces TSBP’s iterative EMD matching with label propagation on *k*-nearest neighbor similarity graphs and introduces adaptive per-class thresholding. These changes address limitations of the original approach and yield improvements in refinement quality and computational scaling. Overall, TGSP matched or exceeded TSBP in almost all test cases, demonstrating broader applicability across datasets and model architectures. By removing the K-means clustering, TGSP also eliminates a dataset-specific hyperparameter while maintaining or improving performance. The graph-based propagation substation of multi-round EMD matching offers smoothness and consistency whilst also scaling better computationally. Although we used fixed hyperparameters in our experiments, dataset-specific and model-specific tuning of these parameters may further improve results.

Future research should focus on several areas to advance the automated analysis of histopathological images. First, the investigation of learning approaches to address rare pathologies where only limited data can be obtained, such as few-shot and zero-shot learning. Our results with YOLOE and YOLO-World suggest that prompt-guided and open-vocabulary approaches show promise for these scenarios. Second, the development of explainable AI techniques specifically designed for histopathological applications is essential for clinical acceptance and approval. Visualization of feature importance, decision boundaries, and inner mechanisms of models would help pathologists understand and trust the automated systems. Our analyses provide a starting point, but clinical deployment requires extensive validation beyond standardized datasets.

In conclusion, while recent advances in object detection architectures have provided powerful tools for histopathological image analysis, careful consideration of specific application requirements, dataset characteristics, and practical deployment constraints are required for clinical implementation. These findings provide guidance for researchers and clinicians for the development of automated histopathological analysis systems and establish performance benchmarks for future innovations in this area.
