# Supplementary material for: Object detection in histology: A multi-dataset benchmark and test-time inference
Source: PLoS One. 2026 Jul 29;21(7):e0354618. doi: 10.1371/journal.pone.0354618 (PMC13419184; doi:10.1371/journal.pone.0354618)
Supplement: Attachment — Submitted filename: Response to Reviewers.docx [file pone.0354618.s002.docx]

Dear Editor(s),

We thank the reviewers for taking the time to read our manuscript and present their concerns. We have thoroughly revised the manuscript and addressed the points highlighted by the reviewers. We hope that the manuscript is suitable for publication now.

Response to the reviewers:

## Reviewer #1

Reviewer #1: The study presents a comprehensive benchmarking of state-of-the-art YOLO models (v8 through the recently released v12) alongside alternative architectures like RT-DETR, YOLO-World, and YOLOE in the context of histopathology. The evaluation spans five diverse datasets: BCNB, Nuclei, TNBC, MoNuSAC, and CryoNuSeg. Key findings indicate that YOLOv11 is the most consistent performer across various organs and tissue types. Conversely, RT-DETR demonstrated "catastrophic failure" when detecting small objects, which are prevalent in histological images. Beyond the benchmark, the authors propose a novel test-time refinement method called Test-time Graph Similarity Propagation (TGSP). This method uses a k-NN similarity graph and label propagation to re-score detections, outperforming the existing TSBP (Test-time Self-guided Bounding-box Propagation) method by providing better scalability and eliminating certain hyperparameters.
To meet the standards for publication in PLOS One, the following areas require clarification or expansion:

***Reviewer #1 Point 1 -*** In the TNBC dataset analysis, only 11 patients were included, with only one patient used for the test set. How can authors ensure that the high performance (~0.9 mAP) is not a result of "patient-specific" features rather than a true generalized detection of cancerous nuclei?

**Reply:** We have extended our analysis on TNBC by creating an 11-fold evaluation of the YOLO11 model, where each patient sequentially becomes the unseen test subset. The results indicate that regardless of split, the YOLO11 is capable of a high amount of generalization showing an average mAP@50 performance of ~90% with minimum variation of ±3% depending on the split.

| **Model/Metric** | **mAP@50** | **mAP@50-95** | **Mean F1** | **Mean Precision** | **Mean Recall** |
| --- | --- | --- | --- | --- | --- |
| TNBC-01 | 0.929±0.001 | 0.619±0.018 | 0.886±0.002 | 0.889±0.012 | 0.883±0.007 |
| TNBC-02 | 0.928±0.006 | 0.630±0.008 | 0.891±0.006 | 0.930±0.028 | 0.855±0.014 |
| TNBC-03 | **0.936±0.001** | 0.646±0.011 | 0.899±0.003 | 0.908±0.016 | **0.891±0.021** |
| TNBC-04 | 0.868±0.007 | 0.594±0.002 | 0.835±0.012 | 0.865±0.013 | 0.806±0.011 |
| TNBC-05 | 0.894±0.007 | 0.568±0.009 | 0.858±0.020 | 0.845±0.040 | 0.872±0.001 |
| TNBC-06 | 0.866±0.040 | 0.562±0.005 | 0.830±0.018 | 0.847±0.001 | 0.814±0.035 |
| TNBC-07 | 0.925±0.002 | 0.519±0.000 | 0.901±0.008 | 0.919±0.013 | 0.883±0.003 |
| TNBC-08 | 0.891±0.003 | 0.577±0.017 | 0.831±0.010 | 0.824±0.020 | 0.839±0.000 |
| TNBC-09 | 0.913±0.021 | 0.639±0.017 | 0.864±0.014 | 0.889±0.014 | 0.841±0.014 |
| TNBC-10 | 0.866±0.021 | 0.587±0.017 | 0.838±0.022 | 0.867±0.020 | 0.813±0.058 |
| TNBC-11 | 0.926±0.004 | **0.677±0.006** | **0.904±0.005** | **0.937±0.001** | 0.874±0.009 |

***Reviewer #1 Point 2 –*** Authors converted JSON polygons and binary masks into YOLO bounding boxes. In dense clusters (like those in Figure 10), did this conversion lead to significant overlap or "box merging" that might have confused the single-stage detectors during training?

**Reply:** We have added a new analysis to the manuscript comparing all bounding boxes vs only those that do not contain heavy overlap or inclusion (as shown in the Figure below). The results indicate that the significant overlap may actually slightly help the model in detection. Thus, no confusion has occurred.

| **Model/Metric** | **mAP@50** | **mAP@50-95** | **Mean F1** | **Mean Precision** | **Mean Recall** |
| --- | --- | --- | --- | --- | --- |
| Bounding boxes with no heavy overlap or inclusion | 0.724±0.081 | 0.424±0.089 | 0.629±0.151 | 0.552±0.222 | 0.766±0.002 |
| All bounding boxes | **0.784±0.003** | **0.503±0.004** | **0.756±0.007** | **0.745±0.026** | **0.768±0.013** |

***Reviewer #1 Point 3 –*** RT-DETR achieved 0 performance on several datasets. Beyond "small object size," is there a technical reason related to the transformer's fixed-size patch embedding or the lack of a multi-scale feature pyramid that caused this specific architecture to fail where CNN-based YOLOs succeeded?

**Reply:** We thank the reviewer for this question. We would like to clarify that RT-DETR is not a pure fixed-patch Vision Transformer detector; in its design [1], features from the last three backbone stages are passed to a hybrid encoder that performs intra-scale interaction and CNN-based cross-scale feature fusion, and the decoder is initialized from a fixed number of selected encoder features. Therefore, the zero-AP cases in our experiments are unlikely to be explained by a fixed patch embedding alone or by a complete absence of multi-scale processing, because RT-DETR already incorporates multi-scale features. In particular, the combination of limited spatial detail at small object scales and the model’s fixed query initialization can make RT-DETR less robust than CNN-based YOLO variants in highly crowded scenes with many objects; this is a known limitation and there have been attempts at addressing this issue [2]. We already mention in the manuscript:

“RT-DETR replaces the standard Transformer encoder with a hybrid encoder to process intra-scale interactions and cross-scale feature fusion separately”

[1] Y. Zhao et al., "DETRs Beat YOLOs on Real-time Object Detection," 2024 IEEE/CVF Conference on Computer Vision and Pattern Recognition (CVPR), Seattle, WA, USA, 2024, pp. 16965-16974, doi: 10.1109/CVPR52733.2024.01605.

[2] Madan M, Reich C. Strengthening Small Object Detection in Adapted RT-DETR Through Robust Enhancements. *Electronics*. 2025; 14(19):3830. https://doi.org/10.3390/electronics14193830

***Reviewer #1 Point 4 –*** Authors mention that 8x downsampling is often optimal, yet authors used 20x for BCNB. Did authors perform any ablation studies to see if a lower downsampling rate would resolve the "missed detection" failure modes observed in the BCNB benchmark?

**Reply:** We have added a new analysis including different levels of downsampling:

“The impact of downsampling was analyzed with various amounts on the YOLO11 model for the BCNB dataset. The performance for each level of downsampling is shown in Table 3 indicating that more downsampling (x40) would reduce the performance of the model, whilst less downsampling (x8, x16) may bring a slight increase in performance. Nevertheless, with lower downsampling the images have a higher resolution requiring a bigger model which has a higher training and inference time. Therefore, the reduced performance obtained by all models on this dataset cannot be attributed to the downsampling.

Table 3 – Results of YOLO11 object detection model on the BCNB [40] dataset (across 5 runs presented as mean±standard deviation, bold values indicate the best performance for each criterion).

| **Down**  **sampling** | **mAP@50** | **mAP@50-95** | **Mean F1** | **Mean Precision** | **Mean Recall** | **Inference Time [ms]** |
| --- | --- | --- | --- | --- | --- | --- |
| x8 | 0.268±0.003 | 0.149±0.004 | 0.262±0.005 | 0.404±0.004 | 0.194±0.005 | 207.6 |
| x16 | 0.263±0.002 | 0.148±0.006 | 0.268±0.006 | 0.392±0.005 | 0.204±0.008 | 172.5 |
| x20 | 0.261±0.006 | 0.142±0.005 | 0.242±0.006 | 0.405±0.007 | 0.172±0.005 | 148.2 |
| x40 | 0.216±0.016 | 0.109±0.011 | 0.128±0.012 | 0.385±0.034 | 0.077±0.010 | 121.4 |

”

***Reviewer #1 Point 5 –*** Authors state TGSP has a time complexity of O(N log N * d) for graph construction. In a clinical setting with a Whole Slide Image (WSI) containing thousands of nuclei, what is the actual wall-clock time added by TGSP per image compared to the base model inference?

**Reply:** Both TSBP and TGSP use deep features obtained from a ResNet-50 pretrained model which renders the processing time dependent upon the hardware of the system (CPU or GPU and of course their models). As such, both add a considerable amount of time to the inference. These approaches can only be used as an offline post-processing step as they do not offer real-time performance. We have updated the manuscript to include this information:

“Both TSBP and TGSP use deep features obtained from a ResNet-50 pretrained model which renders their processing time dependent upon the hardware of the system. As such, both add a considerable amount of time to the inference. These approaches can only be used as an offline post-processing step as they do not offer real-time performance.”

***Reviewer #1 Point 6 –*** Authors’ failure mode analysis (Figure 12) shows YOLOv12 has a significantly higher number of background false positives than YOLOv11. Why do authors believe the new "attention-centric" architecture is more prone to identifying non-pathological background as objects?

**Reply:** We thank the reviewer for the comment. The high number of false positives for the YOLOv12 occurs only on the nuclei dataset and is comparable to that of the other models across the other four datasets. We therefore do not think this reflects a general tendency of the attention-centric architecture to mistake background for objects. We have clarified in the revised manuscript that this is an isolated, dataset-specific failure mode rather than a systematic issue as follows:

“YOLO12 also had a large number of background false positives but it compensated with the lowest number of missed detections. Specifically, the high number of background false positives observed for YOLOv12 are confined to the nuclei dataset and should be interpreted as a dataset-specific failure case rather than a general limitation of the proposed attention-centric design.”

***Reviewer #1 Point 7 –*** TGSP uses adaptive per-class quantile thresholds. How does the method perform if the test set is highly imbalanced (e.g., 99% background and 1% target objects)? Would the quantile approach still be able to define a valid "high-confidence" set?

**Reply:** In the traditional object detection approach, there is no background class. Yes, even if there are only a few detections or even just one. Even when including a background class, the per-class threshold is the better approach, as it guarantees that detections are included from all classes. This is one of the reasons for its introduction. We have updated the manuscript to include this information:

“Moreover, the per-class approach allows for a fair partition when class imbalanced detections (such as a class with a low number of detections per image and another with a high number) are found as each class will receive high-confidence and low-confidence predictions.”

***Reviewer #1 Point 8 –*** 8. To further strengthen the contextual framework and clinical relevance of this study, it is recommended to incorporate the following references into the Introduction and Discussion sections. Chou et al. (2024) provides a critical precedent for evaluating YOLO frameworks in cancer detection, directly supporting the methodology of this benchmark. Additionally, because a significant portion of the analyzed data involves breast cancer histology (BCNB, Nuclei, and TNBC), citing Hsu et al. (2025) would provide a modern review of imaging innovations specific to breast cancer diagnosis. Finally, the inclusion of Lai et al. (2024) would offer the reader a broader understanding of the challenges regarding the clinical adoption of machine learning tools in tissue diagnostics, further highlighting the importance of the computational efficiency metrics established in this evaluation.

Recommended Citations:

Chou, C. K., et al. (2024). "Evaluation of Spectrum-Aided Visual Enhancer (SAVE) in Esophageal Cancer Detection Using YOLO Frameworks." Diagnostics.

Hsu, H., et al. (2025). "From Innovation to Application: Can Emerging Imaging Techniques Transform Breast Cancer Diagnosis?" Diagnostics.

Lai, C. L., et al. (2024). "Advancing Hyperspectral Imaging and Machine Learning Tools Towards Clinical Adoption in Tissue Diagnostics: A Comprehensive Review." APL Bioengineering.

**Reply:** We have updated the manuscript to include a short summary of the suggested citations, it now includes the following:

“YOLOv5 and YOLOv8 were evaluated using Spectrum-Aided Visual Enhancer (SAVE) for early esophageal cancer detection by converting white-light images into hyperspectral narrowband-like images and then training on paired white-light and HSI-NBI datasets [28]; YOLOv5 was found to have the best performance through a comparison through precision, recall, F1-score, mAP, and confusion matrices [28]. YOLOv5 was found to have a strong performance for breast-lesion identification and classification for a Contrast-Enhanced Mammography study involving 1,673 patients and 7,443 images [29]. In a YOLOv5-based hyperspectral imaging study [30] for skin-cancer detection, it was shown that the hyperspectral narrowband images (HSI-NBI) can improve the recall for certain classes over the classical RGB images. YOLO-family models are also being explored in combination with spectral or contrast-enhanced imaging inputs [31], [32] to improve detection and classification on the road towards clinical applicability.”

## Reviewer #2

Reviewer #2: In this research is presented a comparative analysis for object detection tasks in histological images of the YOLO architectures, (YOLOv8 to YOLOv12), alongside with alternative architectures including RT-DETR, YOLO-World, and YOLOE across five histology datasets: BCNB, Nuclei, TNBC, MoNuSAC, and CryoNuSeg, The research is interesting in form of the robustness of the analysed architectures and the research application is high in practice. There are still some recommendation to the authors in order to improve paper readability.

***Reviewer #2 Point 1 -*** That recommendation is considered that authors should highlight the best results through the used architectures in the tables (Tables 2 to 6). In that manner, it would be improved the paper readability.

**Reply:** We thank the reviewer for highlighting this small issue. We have updated all the tables to include boldfaced values to highlight the best performance obtained for each criterion.

Finally, we would like to thank the editor and the reviewers for their insightful and helpful comments!
