# Supplementary material for: Object detection in histology: A multi-dataset benchmark and test-time inference
Source: PLoS One. 2026 Jul 29;21(7):e0354618. doi: 10.1371/journal.pone.0354618 (PMC13419184; doi:10.1371/journal.pone.0354618)
Supplement: Peer review history

# Peer review: Decision Letter 0

**DOI:** 10.1371/journal.pone.0354618.r001

**Authors:** Yile Chen

24 Mar 2026

PONE-D-26-06622

Object detection in histology: A multi-dataset benchmark and test-time inference

PLOS One

Dear Dr. Ardelean,

Thank you for submitting your manuscript to PLOS ONE. After careful consideration, we feel that it has merit but does not fully meet PLOS ONE’s publication criteria as it currently stands. Therefore, we invite you to submit a revised version of the manuscript that addresses the points raised during the review process.

Please submit your revised manuscript by May 08 2026 11:59PM. If you will need more time than this to complete your revisions, please reply to this message or contact the journal office at plosone@plos.org. When you're ready to submit your revision, log on to https://www.editorialmanager.com/pone/ and select the 'Submissions Needing Revision' folder to locate your manuscript file.

Please include the following items when submitting your revised manuscript:

- A letter that responds to each point raised by the academic editor and reviewer(s). You should upload this letter as a separate file labeled 'Response to Reviewers'.
- A marked-up copy of your manuscript that highlights changes made to the original version. You should upload this as a separate file labeled 'Revised Manuscript with Track Changes'.
- An unmarked version of your revised paper without tracked changes. You should upload this as a separate file labeled 'Manuscript'.

-->If you would like to make changes to your financial disclosure, please include your updated statement in your cover letter. Guidelines for resubmitting your figure files are available below the reviewer comments at the end of this letter.

If applicable, we recommend that you deposit your laboratory protocols in protocols.io to enhance the reproducibility of your results. Protocols.io assigns your protocol its own identifier (DOI) so that it can be cited independently in the future. For instructions see: https://journals.plos.org/plosone/s/submission-guidelines#loc-laboratory-protocols. Additionally, PLOS ONE offers an option for publishing peer-reviewed Lab Protocol articles, which describe protocols hosted on protocols.io. Read more information on sharing protocols at https://plos.org/protocols?utm_medium=editorial-email&utm_source=authorletters&utm_campaign=protocols.

We look forward to receiving your revised manuscript.

Kind regards,

Yile Chen, Ph.D. in Architecture

Academic Editor

PLOS One

Journal requirements:

When submitting your revision, we need you to address these additional requirements.

1. Please ensure that your manuscript meets PLOS ONE's style requirements, including those for file naming. The PLOS ONE style templates can be found at

https://journals.plos.org/plosone/s/file?id=wjVg/PLOSOne_formatting_sample_main_body.pdf and

https://journals.plos.org/plosone/s/file?id=ba62/PLOSOne_formatting_sample_title_authors_affiliations.pdf

2. Please note that PLOS One has specific guidelines on code sharing for submissions in which author-generated code underpins the findings in the manuscript. In these cases, we expect all author-generated code to be made available without restrictions upon publication of the work. Please review our guidelines at https://journals.plos.org/plosone/s/materials-and-software-sharing#loc-sharing-code and ensure that your code is shared in a way that follows best practice and facilitates reproducibility and reuse.

3. Thank you for stating the following financial disclosure:

“This work is supported by the project "Romanian Hub for Artificial Intelligence-HRIA", Smart Growth, Digitization and Financial Instruments Program, MySMIS no. 334906.”

Please state what role the funders took in the study.  If the funders had no role, please state: "The funders had no role in study design, data collection and analysis, decision to publish, or preparation of the manuscript."

If this statement is not correct you must amend it as needed.

Please include this amended Role of Funder statement in your cover letter; we will change the online submission form on your behalf.

4. Thank you for stating the following in the Funding Section of your manuscript:

“This work is supported by the project "Romanian Hub for Artificial Intelligence-HRIA", Smart Growth, Digitization and Financial Instruments Program, MySMIS no. 334906.”

We note that you have provided funding information that is currently declared in your Funding Statement. However, funding information should not appear in the Acknowledgments section or other areas of your manuscript. We will only publish funding information present in the Funding Statement section of the online submission form.

Please remove any funding-related text from the manuscript and let us know how you would like to update your Funding Statement. Currently, your Funding Statement reads as follows:

“This work is supported by the project "Romanian Hub for Artificial Intelligence-HRIA", Smart Growth, Digitization and Financial Instruments Program, MySMIS no. 334906.”

Please include your amended statements within your cover letter; we will change the online submission form on your behalf.

If the reviewer comments include a recommendation to cite specific previously published works, please review and evaluate these publications to determine whether they are relevant and should be cited. There is no requirement to cite these works unless the editor has indicated otherwise.

Please review your reference list to ensure that it is complete and correct. If you have cited papers that have been retracted, please include the rationale for doing so in the manuscript text, or remove these references and replace them with relevant current references. Any changes to the reference list should be mentioned in the rebuttal letter that accompanies your revised manuscript. If you need to cite a retracted article, indicate the article’s retracted status in the References list and also include a citation and full reference for the retraction notice.

[Note: HTML markup is below. Please do not edit.]

Reviewers' comments:

Reviewer's Responses to Questions

**Comments to the Author**

1. Is the manuscript technically sound, and do the data support the conclusions?

The manuscript must describe a technically sound piece of scientific research with data that supports the conclusions. Experiments must have been conducted rigorously, with appropriate controls, replication, and sample sizes. The conclusions must be drawn appropriately based on the data presented.

Reviewer #1: Yes

Reviewer #2: Yes

**********

2. Has the statistical analysis been performed appropriately and rigorously?

Reviewer #1: Yes

Reviewer #2: Yes

**********

3. Have the authors made all data underlying the findings in their manuscript fully available?

The PLOS Data policy requires authors to make all data underlying the findings described in their manuscript fully available without restriction, with rare exception (please refer to the Data Availability Statement in the manuscript PDF file). The data should be provided as part of the manuscript or its supporting information, or deposited to a public repository. For example, in addition to summary statistics, the data points behind means, medians and variance measures should be available. If there are restrictions on publicly sharing data—e.g. participant privacy or use of data from a third party—those must be specified.

Reviewer #1: Yes

Reviewer #2: Yes

**********

4. Is the manuscript presented in an intelligible fashion and written in standard English?

PLOS ONE does not copyedit accepted manuscripts, so the language in submitted articles must be clear, correct, and unambiguous. Any typographical or grammatical errors should be corrected at revision, so please note any specific errors here.

Reviewer #1: Yes

Reviewer #2: Yes

**********

5. Review Comments to the Author

Please use the space provided to explain your answers to the questions above. You may also include additional comments for the author, including concerns about dual publication, research ethics, or publication ethics. (Please upload your review as an attachment if it exceeds 20,000 characters)

Reviewer #1: The study presents a comprehensive benchmarking of state-of-the-art YOLO models (v8 through the recently released v12) alongside alternative architectures like RT-DETR, YOLO-World, and YOLOE in the context of histopathology. The evaluation spans five diverse datasets: BCNB, Nuclei, TNBC, MoNuSAC, and CryoNuSeg. Key findings indicate that YOLOv11 is the most consistent performer across various organs and tissue types. Conversely, RT-DETR demonstrated "catastrophic failure" when detecting small objects, which are prevalent in histological images. Beyond the benchmark, the authors propose a novel test-time refinement method called Test-time Graph Similarity Propagation (TGSP). This method uses a k-NN similarity graph and label propagation to re-score detections, outperforming the existing TSBP (Test-time Self-guided Bounding-box Propagation) method by providing better scalability and eliminating certain hyperparameters.

To meet the standards for publication in PLOS One, the following areas require clarification or expansion:

1. In the TNBC dataset analysis, only 11 patients were included, with only one patient used for the test set. How can authors ensure that the high performance (~0.9 mAP) is not a result of "patient-specific" features rather than a true generalized detection of cancerous nuclei?

2. Authors converted JSON polygons and binary masks into YOLO bounding boxes. In dense clusters (like those in Figure 10), did this conversion lead to significant overlap or "box merging" that might have confused the single-stage detectors during training?

3. RT-DETR achieved 0 performance on several datasets. Beyond "small object size," is there a technical reason related to the transformer's fixed-size patch embedding or the lack of a multi-scale feature pyramid that caused this specific architecture to fail where CNN-based YOLOs succeeded?

4. Authors mention that 8x downsampling is often optimal, yet authors used 20x for BCNB. Did authors perform any ablation studies to see if a lower downsampling rate would resolve the "missed detection" failure modes observed in the BCNB benchmark?

5. Authors state TGSP has a time complexity of O(N log N * d) for graph construction. In a clinical setting with a Whole Slide Image (WSI) containing thousands of nuclei, what is the actual wall-clock time added by TGSP per image compared to the base model inference?

6. Authors’ failure mode analysis (Figure 12) shows YOLOv12 has a significantly higher number of background false positives than YOLOv11. Why do authors believe the new "attention-centric" architecture is more prone to identifying non-pathological background as objects?

7. TGSP uses adaptive per-class quantile thresholds. How does the method perform if the test set is highly imbalanced (e.g., 99% background and 1% target objects)? Would the quantile approach still be able to define a valid "high-confidence" set?

8. To further strengthen the contextual framework and clinical relevance of this study, it is recommended to incorporate the following references into the Introduction and Discussion sections. Chou et al. (2024) provides a critical precedent for evaluating YOLO frameworks in cancer detection, directly supporting the methodology of this benchmark. Additionally, because a significant portion of the analyzed data involves breast cancer histology (BCNB, Nuclei, and TNBC), citing Hsu et al. (2025) would provide a modern review of imaging innovations specific to breast cancer diagnosis. Finally, the inclusion of Lai et al. (2024) would offer the reader a broader understanding of the challenges regarding the clinical adoption of machine learning tools in tissue diagnostics, further highlighting the importance of the computational efficiency metrics established in this evaluation.

Recommended Citations:

Chou, C. K., et al. (2024). "Evaluation of Spectrum-Aided Visual Enhancer (SAVE) in Esophageal Cancer Detection Using YOLO Frameworks." Diagnostics.

Hsu, H., et al. (2025). "From Innovation to Application: Can Emerging Imaging Techniques Transform Breast Cancer Diagnosis?" Diagnostics.

Lai, C. L., et al. (2024). "Advancing Hyperspectral Imaging and Machine Learning Tools Towards Clinical Adoption in Tissue Diagnostics: A Comprehensive Review." APL Bioengineering.

Reviewer #2: In this research is presented a comparative analysis for object detection tasks in histological images of the YOLO architectures, (YOLOv8 to YOLOv12), alongside with alternative architectures including RT-DETR, YOLO-World,

and YOLOE across five histology datasets: BCNB, Nuclei, TNBC, MoNuSAC, and CryoNuSeg, The research is interesting in form of the robustness of the analysed architectures and the research application is high in practice. There are still some recommendation to the authors in order to improve paper readability. That recommendation is considered that authors should highlight the best results through the used architectures in the tables (Tables 2 to 6). In that manner, it would be improved the paper readability.

**********

6. PLOS authors have the option to publish the peer review history of their article (what does this mean?). If published, this will include your full peer review and any attached files.

If you choose “no”, your identity will remain anonymous but your review may still be made public.

**Do you want your identity to be public for this peer review?**  For information about this choice, including consent withdrawal, please see our Privacy Policy.

Reviewer #1: No

Reviewer #2: No

**********

[NOTE: If reviewer comments were submitted as an attachment file, they will be attached to this email and accessible via the submission site. Please log into your account, locate the manuscript record, and check for the action link "View Attachments". If this link does not appear, there are no attachment files.]

To ensure your figures meet our technical requirements, please review our figure guidelines: https://journals.plos.org/plosone/s/figures

You may also use PLOS’s free figure tool, NAAS, to help you prepare publication quality figures: https://journals.plos.org/plosone/s/figures#loc-tools-for-figure-preparation.

NAAS will assess whether your figures meet our technical requirements by comparing each figure against our figure specifications.

# Author comment: Author response to Decision Letter 1

**DOI:** 10.1371/journal.pone.0354618.r002

6 Apr 2026

Dear Editor(s),

We thank the reviewers for taking the time to read our manuscript and present their concerns. We have thoroughly revised the manuscript and addressed the points highlighted by the reviewers. We hope that the manuscript is suitable for publication now.

Response to the reviewers:

Reviewer #1

Reviewer #1: The study presents a comprehensive benchmarking of state-of-the-art YOLO models (v8 through the recently released v12) alongside alternative architectures like RT-DETR, YOLO-World, and YOLOE in the context of histopathology. The evaluation spans five diverse datasets: BCNB, Nuclei, TNBC, MoNuSAC, and CryoNuSeg. Key findings indicate that YOLOv11 is the most consistent performer across various organs and tissue types. Conversely, RT-DETR demonstrated "catastrophic failure" when detecting small objects, which are prevalent in histological images. Beyond the benchmark, the authors propose a novel test-time refinement method called Test-time Graph Similarity Propagation (TGSP). This method uses a k-NN similarity graph and label propagation to re-score detections, outperforming the existing TSBP (Test-time Self-guided Bounding-box Propagation) method by providing better scalability and eliminating certain hyperparameters.

To meet the standards for publication in PLOS One, the following areas require clarification or expansion:

Reviewer #1 Point 1 - In the TNBC dataset analysis, only 11 patients were included, with only one patient used for the test set. How can authors ensure that the high performance (~0.9 mAP) is not a result of "patient-specific" features rather than a true generalized detection of cancerous nuclei?

Reply: We have extended our analysis on TNBC by creating an 11-fold evaluation of the YOLO11 model, where each patient sequentially becomes the unseen test subset. The results indicate that regardless of split, the YOLO11 is capable of a high amount of generalization showing an average mAP@50 performance of ~90% with minimum variation of ±3% depending on the split.

Model/Metric mAP@50 mAP@50-95 Mean F1 Mean Precision Mean Recall

TNBC-01 0.929±0.001 0.619±0.018 0.886±0.002 0.889±0.012 0.883±0.007

TNBC-02 0.928±0.006 0.630±0.008 0.891±0.006 0.930±0.028 0.855±0.014

TNBC-03 0.936±0.001 0.646±0.011 0.899±0.003 0.908±0.016 0.891±0.021

TNBC-04 0.868±0.007 0.594±0.002 0.835±0.012 0.865±0.013 0.806±0.011

TNBC-05 0.894±0.007 0.568±0.009 0.858±0.020 0.845±0.040 0.872±0.001

TNBC-06 0.866±0.040 0.562±0.005 0.830±0.018 0.847±0.001 0.814±0.035

TNBC-07 0.925±0.002 0.519±0.000 0.901±0.008 0.919±0.013 0.883±0.003

TNBC-08 0.891±0.003 0.577±0.017 0.831±0.010 0.824±0.020 0.839±0.000

TNBC-09 0.913±0.021 0.639±0.017 0.864±0.014 0.889±0.014 0.841±0.014

TNBC-10 0.866±0.021 0.587±0.017 0.838±0.022 0.867±0.020 0.813±0.058

TNBC-11 0.926±0.004 0.677±0.006 0.904±0.005 0.937±0.001 0.874±0.009

Reviewer #1 Point 2 – Authors converted JSON polygons and binary masks into YOLO bounding boxes. In dense clusters (like those in Figure 10), did this conversion lead to significant overlap or "box merging" that might have confused the single-stage detectors during training?

Reply: We have added a new analysis to the manuscript comparing all bounding boxes vs only those that do not contain heavy overlap or inclusion (as shown in the Figure below). The results indicate that the significant overlap may actually slightly help the model in detection. Thus, no confusion has occurred.

Model/Metric mAP@50 mAP@50-95 Mean F1 Mean Precision Mean Recall

Bounding boxes with no heavy overlap or inclusion 0.724±0.081 0.424±0.089 0.629±0.151 0.552±0.222 0.766±0.002

All bounding boxes 0.784±0.003 0.503±0.004 0.756±0.007 0.745±0.026 0.768±0.013

Reviewer #1 Point 3 – RT-DETR achieved 0 performance on several datasets. Beyond "small object size," is there a technical reason related to the transformer's fixed-size patch embedding or the lack of a multi-scale feature pyramid that caused this specific architecture to fail where CNN-based YOLOs succeeded?

Reply: We thank the reviewer for this question. We would like to clarify that RT-DETR is not a pure fixed-patch Vision Transformer detector; in its design [1], features from the last three backbone stages are passed to a hybrid encoder that performs intra-scale interaction and CNN-based cross-scale feature fusion, and the decoder is initialized from a fixed number of selected encoder features. Therefore, the zero-AP cases in our experiments are unlikely to be explained by a fixed patch embedding alone or by a complete absence of multi-scale processing, because RT-DETR already incorporates multi-scale features. In particular, the combination of limited spatial detail at small object scales and the model’s fixed query initialization can make RT-DETR less robust than CNN-based YOLO variants in highly crowded scenes with many objects; this is a known limitation and there have been attempts at addressing this issue [2]. We already mention in the manuscript:

“RT-DETR replaces the standard Transformer encoder with a hybrid encoder to process intra-scale interactions and cross-scale feature fusion separately”

[1] Y. Zhao et al., "DETRs Beat YOLOs on Real-time Object Detection," 2024 IEEE/CVF Conference on Computer Vision and Pattern Recognition (CVPR), Seattle, WA, USA, 2024, pp. 16965-16974, doi: 10.1109/CVPR52733.2024.01605.

[2] Madan M, Reich C. Strengthening Small Object Detection in Adapted RT-DETR Through Robust Enhancements. Electronics. 2025; 14(19):3830. https://doi.org/10.3390/electronics14193830

Reviewer #1 Point 4 – Authors mention that 8x downsampling is often optimal, yet authors used 20x for BCNB. Did authors perform any ablation studies to see if a lower downsampling rate would resolve the "missed detection" failure modes observed in the BCNB benchmark?

Reply: We have added a new analysis including different levels of downsampling:

“The impact of downsampling was analyzed with various amounts on the YOLO11 model for the BCNB dataset. The performance for each level of downsampling is shown in Table 3 indicating that more downsampling (x40) would reduce the performance of the model, whilst less downsampling (x8, x16) may bring a slight increase in performance. Nevertheless, with lower downsampling the images have a higher resolution requiring a bigger model which has a higher training and inference time. Therefore, the reduced performance obtained by all models on this dataset cannot be attributed to the downsampling.

Table 3 – Results of YOLO11 object detection model on the BCNB [40] dataset (across 5 runs presented as mean±standard deviation, bold values indicate the best performance for each criterion).

Down

sampling mAP@50 mAP@50-95 Mean F1 Mean Precision Mean Recall Inference Time [ms]

x8 0.268±0.003 0.149±0.004 0.262±0.005 0.404±0.004 0.194±0.005 207.6

x16 0.263±0.002 0.148±0.006 0.268±0.006 0.392±0.005 0.204±0.008 172.5

x20 0.261±0.006 0.142±0.005 0.242±0.006 0.405±0.007 0.172±0.005 148.2

x40 0.216±0.016 0.109±0.011 0.128±0.012 0.385±0.034 0.077±0.010 121.4

”Reviewer #1 Point 5 – Authors state TGSP has a time complexity of O(N log N * d) for graph construction. In a clinical setting with a Whole Slide Image (WSI) containing thousands of nuclei, what is the actual wall-clock time added by TGSP per image compared to the base model inference?

Reply: Both TSBP and TGSP use deep features obtained from a ResNet-50 pretrained model which renders the processing time dependent upon the hardware of the system (CPU or GPU and of course their models). As such, both add a considerable amount of time to the inference. These approaches can only be used as an offline post-processing step as they do not offer real-time performance. We have updated the manuscript to include this information:

“Both TSBP and TGSP use deep features obtained from a ResNet-50 pretrained model which renders their processing time dependent upon the hardware of the system. As such, both add a considerable amount of time to the inference. These approaches can only be used as an offline post-processing step as they do not offer real-time performance.”

Reviewer #1 Point 6 – Authors’ failure mode analysis (Figure 12) shows YOLOv12 has a significantly higher number of background false positives than YOLOv11. Why do authors believe the new "attention-centric" architecture is more prone to identifying non-pathological background as objects?

Reply: We thank the reviewer for the comment. The high number of false positives for the YOLOv12 occurs only on the nuclei dataset and is comparable to that of the other models across the other four datasets. We therefore do not think this reflects a general tendency of the attention-centric architecture to mistake background for objects. We have clarified in the revised manuscript that this is an isolated, dataset-specific failure mode rather than a systematic issue as follows:

“YOLO12 also had a large number of background false positives but it compensated with the lowest number of missed detections. Specifically, the high number of background false positives observed for YOLOv12 are confined to the nuclei dataset and should be interpreted as a dataset-specific failure case rather than a general limitation of the proposed attention-centric design.”

Reviewer #1 Point 7 – TGSP uses adaptive per-class quantile thresholds. How does the method perform if the test set is highly imbalanced (e.g., 99% background and 1% target objects)? Would the quantile approach still be able to define a valid "high-confidence" set?

Reply: In the traditional object detection approach, there is no background class. Yes, even if there are only a few detections or even just one. Even when including a background class, the per-class threshold is the better approach, as it guarantees that detections are included from all classes. This is one of the reasons for its introduction. We have updated the manuscript to include this information:

“Moreover, the per-class approach allows for a fair partition when class imbalanced detections (such as a class with a low number of detections per image and another with a high number) are found as each class will receive high-confidence and low-confidence predictions.”

Reviewer #1 Point 8 – 8. To further strengthen the contextual framework and clinical relevance of this study, it is recommended to incorporate the following references into the Introduction and Discussion sections. Chou et al. (2024) provides a critical precedent for evaluating YOLO frameworks in cancer detection, directly supporting the methodology of this benchmark. Additionally, because a significant portion of the analyzed data involves breast cancer histology (BCNB, Nuclei, and TNBC), citing Hsu et al. (2025) would provide a modern review of imaging innovations specific to breast cancer diagnosis. Finally, the inclusion of Lai et al. (2024) would offer the reader a broader understanding of the challenges regarding the clinical adoption of machine learning tools in tissue diagnostics, further highlighting the importance of the computational efficiency metrics established in this evaluation.

Recommended Citations:

Chou, C. K., et al. (2024). "Evaluation of Spectrum-Aided Visual Enhancer (SAVE) in Esophageal Cancer Detection Using YOLO Frameworks." Diagnostics.

Hsu, H., et al. (2025). "From Innovation to Application: Can Emerging Imaging Techniques Transform Breast Cancer Diagnosis?" Diagnostics.

Lai, C. L., et al. (2024). "Advancing Hyperspectral Imaging and Machine Learning Tools Towards Clinical Adoption in Tissue Diagnostics: A Comprehensive Review." APL Bioengineering.

Reply: We have updated the manuscript to include a short summary of the suggested citations, it now includes the following:

“YOLOv5 and YOLOv8 were evaluated using Spectrum-Aided Visual Enhancer (SAVE) for early esophageal cancer detection by converting white-light images into hyperspectral narrowband-like images and then training on paired white-light and HSI-NBI datasets [28]; YOLOv5 was found to have the best performance through a comparison through precision, recall, F1-score, mAP, and confusion matrices [28]. YOLOv5 was found to have a strong performance for breast-lesion identification and classification for a Contrast-Enhanced Mammography study involving 1,673 patients and 7,443 images [29]. In a YOLOv5-based hyperspectral imaging study [30] for skin-cancer detection, it was shown that the hyperspectral narrowband images (HSI-NBI) can improve the recall for certain classes over the classical RGB images. YOLO-family models are also being explored in combination with spectral or contrast-enhanced imaging inputs [31], [32] to improve detection and classification on the road towards clinical applicability.”

Reviewer #2

Reviewer #2: In this research is presented a comparative analysis for object detection tasks in histological images of the YOLO architectures, (YOLOv8 to YOLOv12), alongside with alternative architectures including RT-DETR, YOLO-World, and YOLOE across five histology datasets: BCNB, Nuclei, TNBC, MoNuSAC, and CryoNuSeg, The research is interesting in form of the robustness of the analysed architectures and the research application is high in practice. There are still some recommendation to the authors in order to improve paper readability.

Reviewer #2 Point 1 - That recommendation is considered that authors should highlight the best results through the used architectures in the tables (Tables 2 to 6). In that manner, it would be improved the paper readability.

Reply: We thank the reviewer for highlighting this small issue. We have updated all the tables to include boldfaced values to highlight the best performance obtained for each criterion.

Finally, we would like to thank the editor and the reviewers for their insightful and helpful comments!

Attachment Submitted filename: Response to Reviewers.docx

# Peer review: Decision Letter 1

**DOI:** 10.1371/journal.pone.0354618.r003

**Authors:** Yile Chen

9 Jul 2026

Object detection in histology: A multi-dataset benchmark and test-time inference

PONE-D-26-06622R1

Dear Dr. Eugen,

We’re pleased to inform you that your manuscript has been judged scientifically suitable for publication and will be formally accepted for publication once it meets all outstanding technical requirements.

Within one week, you’ll receive an e-mail detailing the required amendments. When these have been addressed, you’ll receive a formal acceptance letter and your manuscript will be scheduled for publication.

An invoice will be generated when your article is formally accepted. Please note, if your institution has a publishing partnership with PLOS and your article meets the relevant criteria, all or part of your publication costs will be covered. Please make sure your user information is up-to-date by logging into Editorial Manager at Editorial Manager® and clicking the ‘Update My Information' link at the top of the page. For questions related to billing, please contact billing support.

If your institution or institutions have a press office, please notify them about your upcoming paper to help maximize its impact. If they’ll be preparing press materials, please inform our press team as soon as possible -- no later than 48 hours after receiving the formal acceptance. Your manuscript will remain under strict press embargo until 2 pm Eastern Time on the date of publication. For more information, please contact onepress@plos.org.

Kind regards,

Yile Chen, Ph.D. in Architecture

Academic Editor

PLOS One

Additional Editor Comments (optional):

Reviewers' comments:

Reviewer's Responses to Questions

**Comments to the Author**

1. If the authors have adequately addressed your comments raised in a previous round of review and you feel that this manuscript is now acceptable for publication, you may indicate that here to bypass the “Comments to the Author” section, enter your conflict of interest statement in the “Confidential to Editor” section, and submit your "Accept" recommendation.

Reviewer #1: All comments have been addressed

Reviewer #2: All comments have been addressed

**********

2. Is the manuscript technically sound, and do the data support the conclusions?

The manuscript must describe a technically sound piece of scientific research with data that supports the conclusions. Experiments must have been conducted rigorously, with appropriate controls, replication, and sample sizes. The conclusions must be drawn appropriately based on the data presented.

Reviewer #1: Yes

Reviewer #2: Yes

**********

3. Has the statistical analysis been performed appropriately and rigorously?

Reviewer #1: Yes

Reviewer #2: Yes

**********

4. Have the authors made all data underlying the findings in their manuscript fully available?

The PLOS Data policy requires authors to make all data underlying the findings described in their manuscript fully available without restriction, with rare exception (please refer to the Data Availability Statement in the manuscript PDF file). The data should be provided as part of the manuscript or its supporting information, or deposited to a public repository. For example, in addition to summary statistics, the data points behind means, medians and variance measures should be available. If there are restrictions on publicly sharing data—e.g. participant privacy or use of data from a third party—those must be specified.

Reviewer #1: Yes

Reviewer #2: Yes

**********

5. Is the manuscript presented in an intelligible fashion and written in standard English?

PLOS ONE does not copyedit accepted manuscripts, so the language in submitted articles must be clear, correct, and unambiguous. Any typographical or grammatical errors should be corrected at revision, so please note any specific errors here.

Reviewer #1: Yes

Reviewer #2: Yes

**********

6. Review Comments to the Author

Please use the space provided to explain your answers to the questions above. You may also include additional comments for the author, including concerns about dual publication, research ethics, or publication ethics. (Please upload your review as an attachment if it exceeds 20,000 characters)

Reviewer #1: This article presents a comprehensive benchmark test of state-of-the-art YOLO models (from v8 to the latest v12) and other alternative architectures (such as RT-DETR, YOLO-World, YOLOE) on five diverse histology datasets (BCNB, Nuclei, TNBC, MoNuSAC, CryoNuSeg). The authors all reply my concerns, this article can be accepted by PLOS One.

Reviewer #2: (No Response)

**********

7. PLOS authors have the option to publish the peer review history of their article (what does this mean?). If published, this will include your full peer review and any attached files.

If you choose “no”, your identity will remain anonymous but your review may still be made public.

**Do you want your identity to be public for this peer review?**  For information about this choice, including consent withdrawal, please see our Privacy Policy.

Reviewer #1: **Yes:** Hsiang-Chen Wang

Reviewer #2: **Yes:** Dimitrije Bujakovic

**********

# Editor report: Acceptance letter

**DOI:** 10.1371/journal.pone.0354618.r004

**Authors:** Yile Chen

PONE-D-26-06622R1

PLOS One

Dear Dr. Ardelean,

I'm pleased to inform you that your manuscript has been deemed suitable for publication in PLOS One. Congratulations! Your manuscript is now being handed over to our production team.

At this stage, our production department will prepare your paper for publication. This includes ensuring the following:

* All references, tables, and figures are properly cited

* All relevant supporting information is included in the manuscript submission,

* There are no issues that prevent the paper from being properly typeset

You will receive further instructions from the production team, including instructions on how to review your proof when it is ready. Please keep in mind that we are working through a large volume of accepted articles, so please give us a few days to review your paper and let you know the next and final steps.

Lastly, if your institution or institutions have a press office, please let them know about your upcoming paper now to help maximize its impact. If they'll be preparing press materials, please inform our press team within the next 48 hours. Your manuscript will remain under strict press embargo until 2 pm Eastern Time on the date of publication. For more information, please contact onepress@plos.org.

You will receive an invoice from PLOS for your publication fee after your manuscript has reached the completed accept phase. If you receive an email requesting payment before acceptance or for any other service, this may be a phishing scheme. Learn how to identify phishing emails and protect your accounts at https://explore.plos.org/phishing.

If we can help with anything else, please email us at customercare@plos.org.

Thank you for submitting your work to PLOS ONE and supporting open access.

Kind regards,

PLOS ONE Editorial Office Staff

on behalf of

Dr. Yile Chen

Academic Editor

PLOS One
